# Supplementary material for: Evaluating large language models for assessment of psychosis risk
Source: NPJ Digit Med. 2026 Jul 23;9:554. doi: 10.1038/s41746-026-02928-4 (PMC13396665; doi:10.1038/s41746-026-02928-4)
Supplement: Supplementary file 1 — Supplementary information [file 41746_2026_2928_MOESM1_ESM.pdf]

Supplementary information: Evaluating Large Language Models for Assessment of Psychosis Risk

Score agreement across model scales

Supplementary Figs. 1–3 summarize predicted versus clinician-rated scores for the remaining models, stratified by model scale (small, medium, and large checkpoints). For each model, scatter plots show item-level predicted scores against clinician ratings for severity and frequency, with the identity line indicating perfect agreement and point size reflecting the number of overlapping observations. The accompanying heatmaps display the joint distribution of predicted and true scores, highlighting where models concentrate mass along the diagonal (good agreement) versus systematic off-diagonal shifts (bias).

Across models, agreement was typically strongest near commonly observed rating levels, and most discrepancies reflected off-by-one differences between adjacent categories, consistent with the ordinal nature of PSYCHS scoring. Smaller models more frequently exhibited upward shifts at lower clinician ratings, consistent with a tendency toward overestimation and reduced specificity in downstream detection, whereas larger models showed tighter clustering around the identity line and more concentrated diagonal structure in the heatmaps. Together, these figures provide a scale-stratified view of calibration and error patterns that complements the aggregated metrics reported in Table 2.

CHR-P detection performance stratified by symptom domain

Supplementary Fig. 4 summarizes domain-stratified CHR-P detection accuracy for three representative models (gemma-3n-E4B-it, Qwen3-30B-A3B-Instruct-2507, and Llama-3.3-70B-Instruct), illustrating how performance varies across symptom domains. Supplementary Tables 1–5 show domain-stratified CHR-P detection performance across the Llama, Qwen, Gemma, MedGemma, and Phi model families. For each symptom domain, we report the number of subject-visits and positive events, alongside accuracy, sensitivity, specificity, F1 score, and MCC. These results were calculated at the subject-visit level using a pragmatic PSYCHS-aligned detection rule derived from the predicted severity and frequency scores, allowing comparison of model performance across different symptom domains and model families.

Fairness diagnostics: demographic parity and equalized odds

Supplementary Tables 6–11 report group-stratified performance to assess fairness-related properties of CHR-P detection across age, gender, ethnicity, first language and recruitment site. We summarise the demographic parity and equalised odds (both for true positive rate [TPR] and false positive rate [FPR]) for each subgroup.

These stratified summaries are intended as diagnostic measures rather than definitive fairness claims. Differences in these metrics can reflect a mixture of factors, including heterogeneity in case mix, transcript completeness, and site-specific interviewing practices, as well as potential model bias. Accordingly, we interpret subgroup deviations as signals motivating further prospective evaluation, including larger samples per subgroup, confidence intervals for group metrics, and clinician-led review of discordant cases to understand sources of disparity before any clinical deployment.

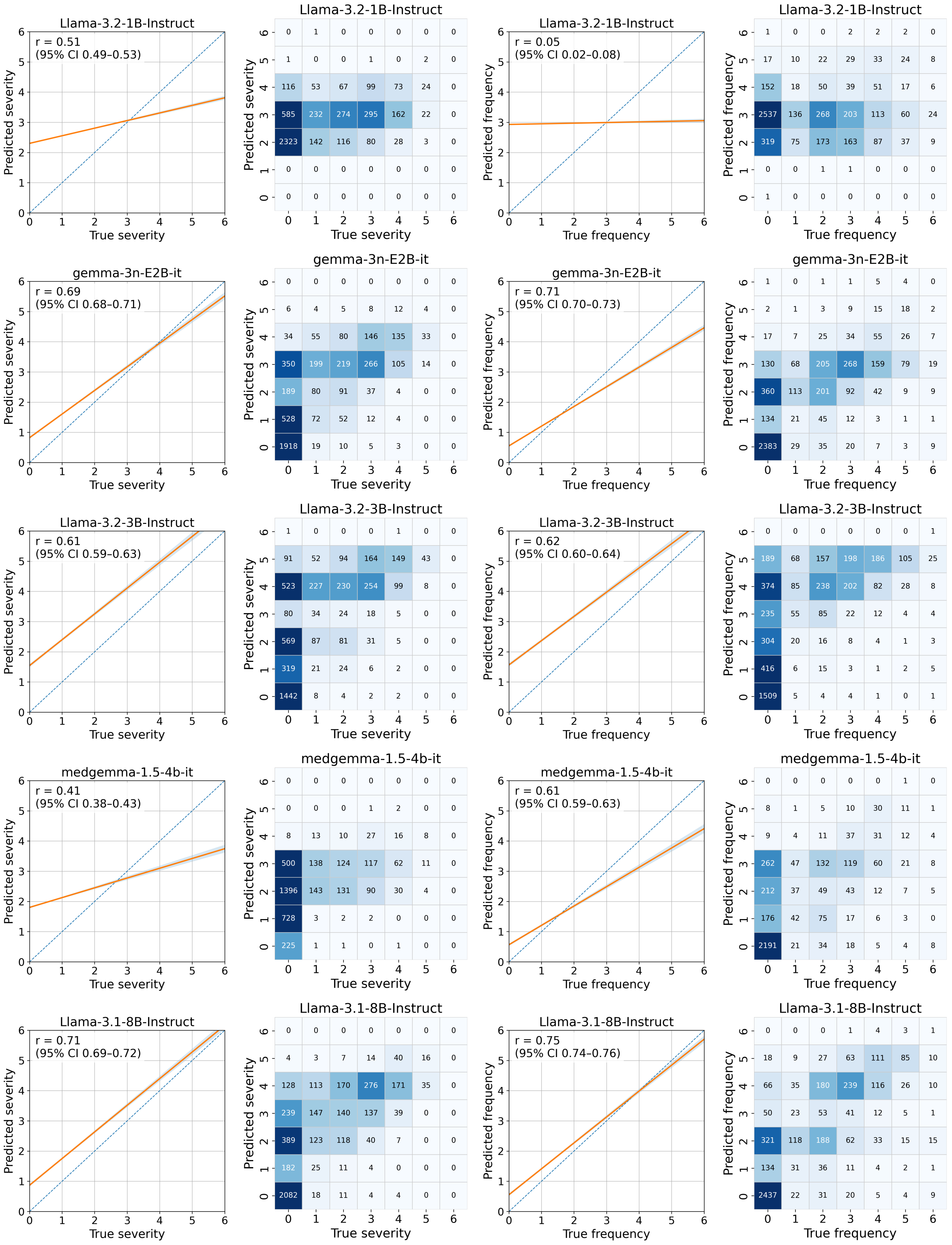

Supplementary Figure 1: Score agreement for small models (< 10B).

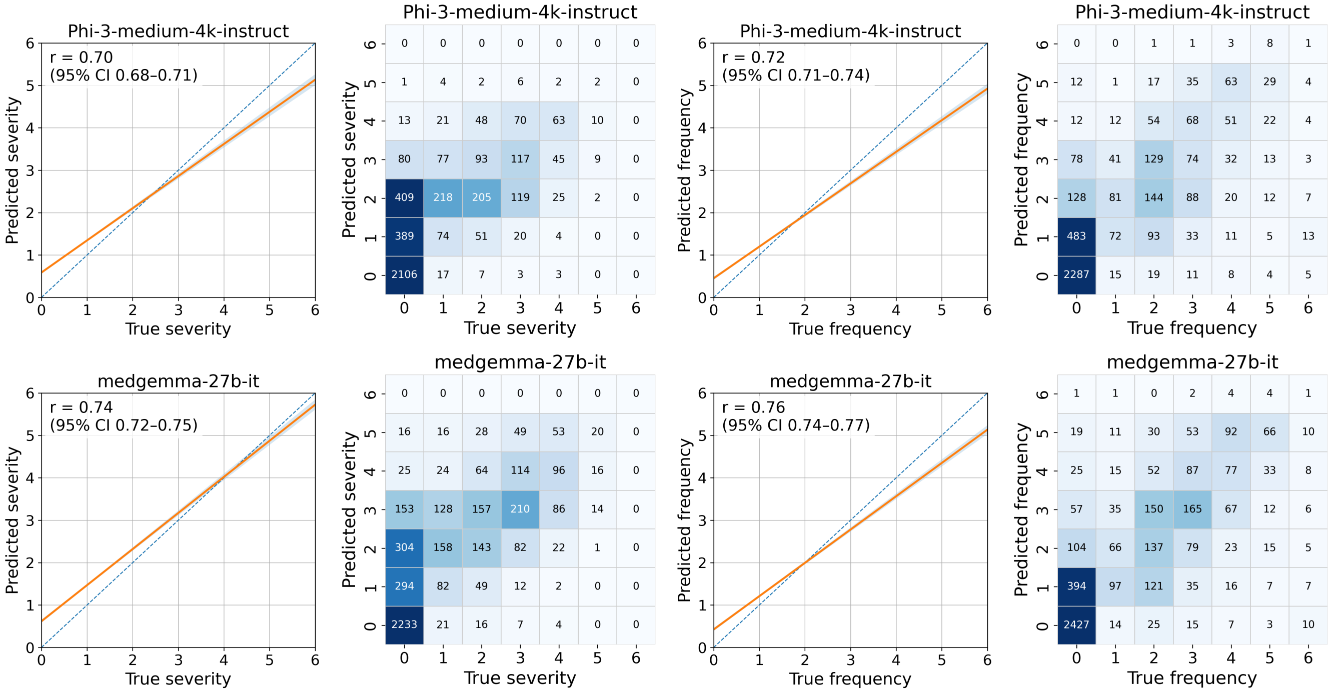

Supplementary Figure 2: Score agreement for medium models (10–30B).

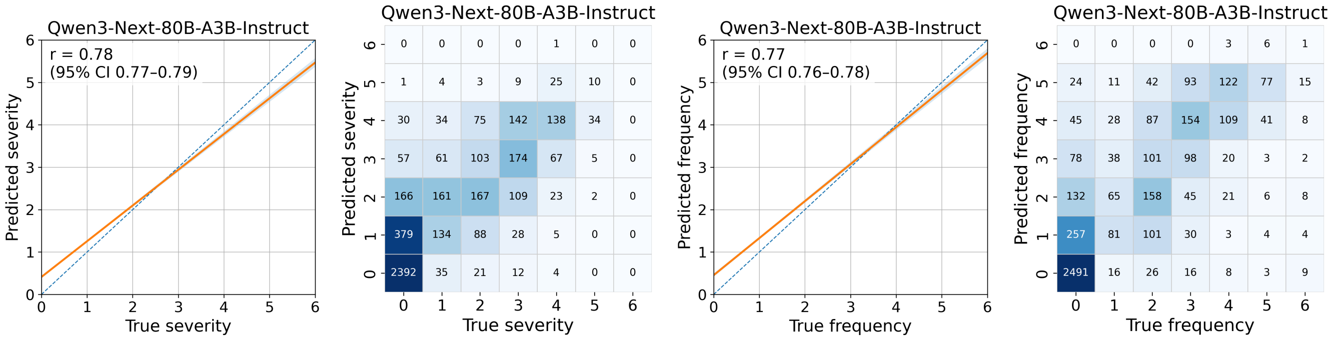

Supplementary Figure 3: Score agreement for large models (> 30B).

Supplementary Table 1: CHR-P detection performance across the Llama family, stratified by symptom domain. Metrics are computed using a pragmatic PSYCHS-aligned gate derived from predicted severity and frequency. Values are proportions.

Model	Symptom Domain	n (Events)	Accuracy	Sensitivity	Specificity	F1	MCC
Llama-3.2-1B-Instruct	Q1	551 (255)	0.613	0.475	0.733	0.532	0.215
Llama-3.2-1B-Instruct	Q2	461 (165)	0.742	0.503	0.875	0.582	0.413
Llama-3.2-1B-Instruct	Q3	424 (38)	0.712	0.868	0.697	0.351	0.338
Llama-3.2-1B-Instruct	Q4	386 (55)	0.728	0.709	0.731	0.426	0.327
Llama-3.2-1B-Instruct	Q5	351 (5)	0.721	0.600	0.723	0.058	0.085
Llama-3.2-1B-Instruct	Q6	334 (7)	0.943	0.286	0.957	0.174	0.163
Llama-3.2-1B-Instruct	Q7	326 (3)	0.712	0.333	0.715	0.021	0.010
Llama-3.2-1B-Instruct	Q8	302 (6)	0.616	0.833	0.611	0.079	0.127
Llama-3.2-1B-Instruct	Q9	261 (26)	0.854	0.692	0.872	0.486	0.437
Llama-3.2-1B-Instruct	Q10	237 (26)	0.886	0.577	0.924	0.526	0.464
Llama-3.2-1B-Instruct	Q11	221 (6)	0.919	0.667	0.926	0.308	0.335
Llama-3.2-1B-Instruct	Q12	218 (0)	0.908	NA	0.908	NA	NA
Llama-3.2-1B-Instruct	Q13	215 (6)	0.921	0.500	0.933	0.261	0.264
Llama-3.2-1B-Instruct	Q14	209 (2)	0.971	0.000	0.981	NA	-0.014
Llama-3.2-1B-Instruct	Q15	195 (10)	0.569	0.700	0.562	0.143	0.116
Llama-3.2-1B-Instruct	Overall	4691 (610)	0.766	0.548	0.799	0.379	0.271
Llama-3.2-1B-Instruct	Subject-level Overall	556 (349)	0.597	0.817	0.227	0.718	0.053
Llama-3.2-3B-Instruct	Q1	551 (255)	0.715	0.961	0.503	0.757	0.511
Llama-3.2-3B-Instruct	Q2	461 (165)	0.623	0.982	0.422	0.651	0.433
Llama-3.2-3B-Instruct	Q3	424 (38)	0.611	0.921	0.580	0.298	0.287
Llama-3.2-3B-Instruct	Q4	386 (55)	0.528	0.964	0.456	0.368	0.300
Llama-3.2-3B-Instruct	Q5	351 (5)	0.675	1.000	0.671	0.081	0.168
Llama-3.2-3B-Instruct	Q6	334 (7)	0.853	0.857	0.853	0.197	0.276
Llama-3.2-3B-Instruct	Q7	326 (3)	0.279	0.667	0.276	0.017	-0.012
Llama-3.2-3B-Instruct	Q8	302 (6)	0.662	0.667	0.662	0.073	0.097
Llama-3.2-3B-Instruct	Q9	261 (26)	0.782	1.000	0.757	0.477	0.487
Llama-3.2-3B-Instruct	Q10	237 (26)	0.743	0.885	0.725	0.430	0.402
Llama-3.2-3B-Instruct	Q11	221 (6)	0.919	1.000	0.916	0.400	0.479
Llama-3.2-3B-Instruct	Q12	218 (0)	0.940	NA	0.940	NA	NA
Llama-3.2-3B-Instruct	Q13	215 (6)	0.744	1.000	0.737	0.179	0.269
Llama-3.2-3B-Instruct	Q14	209 (2)	0.895	1.000	0.894	0.154	0.273
Llama-3.2-3B-Instruct	Q15	195 (10)	0.621	0.800	0.611	0.178	0.184
Llama-3.2-3B-Instruct	Overall	4691 (610)	0.685	0.956	0.644	0.441	0.407
Llama-3.2-3B-Instruct	Subject-level Overall	556 (349)	0.646	1.000	0.048	0.780	0.176
Llama-3.1-8B-Instruct	Q1	551 (255)	0.804	0.863	0.753	0.803	0.616
Llama-3.1-8B-Instruct	Q2	461 (165)	0.764	0.897	0.689	0.731	0.562
Llama-3.1-8B-Instruct	Q3	424 (38)	0.762	0.895	0.749	0.402	0.398
Llama-3.1-8B-Instruct	Q4	386 (55)	0.731	0.891	0.704	0.485	0.428
Llama-3.1-8B-Instruct	Q5	351 (5)	0.855	1.000	0.853	0.164	0.276
Llama-3.1-8B-Instruct	Q6	334 (7)	0.979	0.714	0.985	0.588	0.588
Llama-3.1-8B-Instruct	Q7	326 (3)	0.883	0.333	0.889	0.050	0.067
Llama-3.1-8B-Instruct	Q8	302 (6)	0.940	0.167	0.956	0.100	0.081
Llama-3.1-8B-Instruct	Q9	261 (26)	0.877	0.923	0.872	0.600	0.588
Llama-3.1-8B-Instruct	Q10	237 (26)	0.878	0.769	0.891	0.580	0.535
Llama-3.1-8B-Instruct	Q11	221 (6)	0.955	1.000	0.953	0.545	0.598
Llama-3.1-8B-Instruct	Q12	218 (0)	0.972	NA	0.972	NA	NA
Llama-3.1-8B-Instruct	Q13	215 (6)	0.926	1.000	0.923	0.429	0.502
Llama-3.1-8B-Instruct	Q14	209 (2)	0.967	0.000	0.976	NA	-0.015
Llama-3.1-8B-Instruct	Q15	195 (10)	0.800	0.500	0.816	0.204	0.174
Llama-3.1-8B-Instruct	Overall	4691 (610)	0.856	0.859	0.856	0.608	0.565
Llama-3.1-8B-Instruct	Subject-level Overall	556 (349)	0.745	0.960	0.382	0.825	0.442
Llama-3.3-70B-Instruct	Q1	551 (255)	0.808	0.816	0.801	0.797	0.615
Llama-3.3-70B-Instruct	Q2	461 (165)	0.800	0.806	0.797	0.743	0.586
Llama-3.3-70B-Instruct	Q3	424 (38)	0.835	0.684	0.850	0.426	0.383
Llama-3.3-70B-Instruct	Q4	386 (55)	0.788	0.800	0.785	0.518	0.447
Llama-3.3-70B-Instruct	Q5	351 (5)	0.943	0.200	0.954	0.091	0.085
Llama-3.3-70B-Instruct	Q6	334 (7)	0.979	0.429	0.991	0.462	0.452
Llama-3.3-70B-Instruct	Q7	326 (3)	0.972	0.333	0.978	0.182	0.192
Llama-3.3-70B-Instruct	Q8	302 (6)	0.970	0.167	0.986	0.182	0.167
Llama-3.3-70B-Instruct	Q9	261 (26)	0.946	0.923	0.949	0.774	0.757
Llama-3.3-70B-Instruct	Q10	237 (26)	0.928	0.654	0.962	0.667	0.627
Llama-3.3-70B-Instruct	Q11	221 (6)	0.977	0.833	0.981	0.667	0.670
Llama-3.3-70B-Instruct	Q12	218 (0)	0.991	NA	0.991	NA	NA
Llama-3.3-70B-Instruct	Q13	215 (6)	0.967	0.667	0.976	0.533	0.529
Llama-3.3-70B-Instruct	Q14	209 (2)	0.981	0.000	0.990	NA	-0.010
Llama-3.3-70B-Instruct	Q15	195 (10)	0.841	0.500	0.859	0.244	0.217
Llama-3.3-70B-Instruct	Overall	4691 (610)	0.899	0.774	0.917	0.665	0.615
Llama-3.3-70B-Instruct	Subject-level Overall	556 (349)	0.802	0.934	0.580	0.856	0.568

Supplementary Table 2: CHR-P detection performance across the Qwen family, stratified by symptom domain. Metrics are computed using a pragmatic PSYCHS-aligned gate derived from predicted severity and frequency. Values are proportions.

Model	Symptom Domain	n (Events)	Accuracy	Sensitivity	Specificity	F1	MCC
Qwen3-30B-A3B-Instruct-2507	Q1	551 (255)	0.777	0.929	0.645	0.794	0.591
Qwen3-30B-A3B-Instruct-2507	Q2	461 (165)	0.753	0.885	0.679	0.719	0.541
Qwen3-30B-A3B-Instruct-2507	Q3	424 (38)	0.800	0.763	0.803	0.406	0.375
Qwen3-30B-A3B-Instruct-2507	Q4	386 (55)	0.764	0.836	0.752	0.503	0.437
Qwen3-30B-A3B-Instruct-2507	Q5	351 (5)	0.880	0.800	0.882	0.160	0.242
Qwen3-30B-A3B-Instruct-2507	Q6	334 (7)	0.964	0.857	0.966	0.500	0.537
Qwen3-30B-A3B-Instruct-2507	Q7	326 (3)	0.905	0.333	0.910	0.061	0.080
Qwen3-30B-A3B-Instruct-2507	Q8	302 (6)	0.924	0.167	0.939	0.080	0.061
Qwen3-30B-A3B-Instruct-2507	Q9	261 (26)	0.893	0.962	0.885	0.641	0.635
Qwen3-30B-A3B-Instruct-2507	Q10	237 (26)	0.903	0.846	0.910	0.657	0.625
Qwen3-30B-A3B-Instruct-2507	Q11	221 (6)	0.964	1.000	0.963	0.600	0.642
Qwen3-30B-A3B-Instruct-2507	Q12	218 (0)	0.972	NA	0.972	NA	NA
Qwen3-30B-A3B-Instruct-2507	Q13	215 (6)	0.944	0.833	0.947	0.455	0.490
Qwen3-30B-A3B-Instruct-2507	Q14	209 (2)	0.981	0.500	0.986	0.333	0.345
Qwen3-30B-A3B-Instruct-2507	Q15	195 (10)	0.764	0.500	0.778	0.179	0.145
Qwen3-30B-A3B-Instruct-2507	Overall	4691 (610)	0.862	0.875	0.860	0.622	0.583
Qwen3-30B-A3B-Instruct-2507	Subject-level Overall	556 (349)	0.754	0.983	0.367	0.834	0.477
Qwen3-Next-80B-A3B-Instruct	Q1	551 (255)	0.806	0.827	0.787	0.798	0.613
Qwen3-Next-80B-A3B-Instruct	Q2	461 (165)	0.803	0.788	0.811	0.741	0.585
Qwen3-Next-80B-A3B-Instruct	Q3	424 (38)	0.844	0.632	0.865	0.421	0.370
Qwen3-Next-80B-A3B-Instruct	Q4	386 (55)	0.785	0.836	0.776	0.526	0.463
Qwen3-Next-80B-A3B-Instruct	Q5	351 (5)	0.923	0.600	0.928	0.182	0.231
Qwen3-Next-80B-A3B-Instruct	Q6	334 (7)	0.982	0.857	0.985	0.667	0.676
Qwen3-Next-80B-A3B-Instruct	Q7	326 (3)	0.948	0.333	0.954	0.105	0.127
Qwen3-Next-80B-A3B-Instruct	Q8	302 (6)	0.964	0.167	0.980	0.154	0.136
Qwen3-Next-80B-A3B-Instruct	Q9	261 (26)	0.923	0.923	0.923	0.706	0.690
Qwen3-Next-80B-A3B-Instruct	Q10	237 (26)	0.928	0.577	0.972	0.638	0.603
Qwen3-Next-80B-A3B-Instruct	Q11	221 (6)	0.959	0.833	0.963	0.526	0.550
Qwen3-Next-80B-A3B-Instruct	Q12	218 (0)	0.977	NA	0.977	NA	NA
Qwen3-Next-80B-A3B-Instruct	Q13	215 (6)	0.953	0.833	0.957	0.500	0.528
Qwen3-Next-80B-A3B-Instruct	Q14	209 (2)	0.981	0.500	0.986	0.333	0.345
Qwen3-Next-80B-A3B-Instruct	Q15	195 (10)	0.841	0.400	0.865	0.205	0.164
Qwen3-Next-80B-A3B-Instruct	Overall	4691 (610)	0.893	0.780	0.909	0.654	0.603
Qwen3-Next-80B-A3B-Instruct	Subject-level Overall	556 (349)	0.793	0.934	0.556	0.850	0.548

Supplementary Table 3: CHR-P detection performance across the Gemma family, stratified by symptom domain. Metrics are computed using a pragmatic PSYCHS-aligned gate derived from predicted severity and frequency. Values are proportions.

Model	Symptom Domain	n (Events)	Accuracy	Sensitivity	Specificity	F1	MCC
gemma-3n-E2B-it	Q1	551 (255)	0.759	0.871	0.662	0.769	0.539
gemma-3n-E2B-it	Q2	461 (165)	0.785	0.818	0.767	0.732	0.565
gemma-3n-E2B-it	Q3	424 (38)	0.778	0.816	0.775	0.397	0.376
gemma-3n-E2B-it	Q4	386 (55)	0.723	0.855	0.701	0.468	0.400
gemma-3n-E2B-it	Q5	351 (5)	0.875	0.400	0.882	0.083	0.102
gemma-3n-E2B-it	Q6	334 (7)	0.928	0.714	0.933	0.294	0.340
gemma-3n-E2B-it	Q7	326 (3)	0.890	0.333	0.895	0.053	0.070
gemma-3n-E2B-it	Q8	302 (6)	0.917	0.500	0.926	0.194	0.216
gemma-3n-E2B-it	Q9	261 (26)	0.920	0.885	0.923	0.687	0.665
gemma-3n-E2B-it	Q10	237 (26)	0.890	0.577	0.929	0.536	0.475
gemma-3n-E2B-it	Q11	221 (6)	0.941	1.000	0.940	0.480	0.545
gemma-3n-E2B-it	Q12	218 (0)	0.972	NA	0.972	NA	NA
gemma-3n-E2B-it	Q13	215 (6)	0.907	0.667	0.914	0.286	0.315
gemma-3n-E2B-it	Q14	209 (2)	0.967	0.000	0.976	NA	-0.015
gemma-3n-E2B-it	Q15	195 (10)	0.749	0.700	0.751	0.222	0.224
gemma-3n-E2B-it	Overall	4691 (610)	0.850	0.821	0.854	0.587	0.537
gemma-3n-E2B-it	Subject-level Overall	556 (349)	0.728	0.966	0.329	0.817	0.405
gemma-3n-E4B-it	Q1	551 (255)	0.775	0.827	0.730	0.773	0.557
gemma-3n-E4B-it	Q2	461 (165)	0.785	0.788	0.784	0.724	0.555
gemma-3n-E4B-it	Q3	424 (38)	0.809	0.763	0.813	0.417	0.387
gemma-3n-E4B-it	Q4	386 (55)	0.780	0.800	0.776	0.509	0.437
gemma-3n-E4B-it	Q5	351 (5)	0.937	0.400	0.945	0.154	0.172
gemma-3n-E4B-it	Q6	334 (7)	0.964	0.714	0.969	0.455	0.473
gemma-3n-E4B-it	Q7	326 (3)	0.960	0.333	0.966	0.133	0.152
gemma-3n-E4B-it	Q8	302 (6)	0.957	0.167	0.973	0.133	0.115
gemma-3n-E4B-it	Q9	261 (26)	0.920	0.885	0.923	0.687	0.665
gemma-3n-E4B-it	Q10	237 (26)	0.924	0.577	0.967	0.625	0.586
gemma-3n-E4B-it	Q11	221 (6)	0.959	1.000	0.958	0.571	0.619
gemma-3n-E4B-it	Q12	218 (0)	0.982	NA	0.982	NA	NA
gemma-3n-E4B-it	Q13	215 (6)	0.935	0.500	0.947	0.300	0.299
gemma-3n-E4B-it	Q14	209 (2)	0.981	0.000	0.990	NA	-0.010
gemma-3n-E4B-it	Q15	195 (10)	0.795	0.600	0.805	0.231	0.218
gemma-3n-E4B-it	Overall	4691 (610)	0.881	0.780	0.896	0.630	0.577
gemma-3n-E4B-it	Subject-level Overall	556 (349)	0.766	0.948	0.459	0.836	0.489

Supplementary Table 4: CHR-P detection performance across the MedGemma family, stratified by symptom domain. Metrics are computed using a pragmatic PSYCHS-aligned gate derived from predicted severity and frequency. Values are proportions.

Model	Symptom Domain	n (Events)	Accuracy	Sensitivity	Specificity	F1	MCC
medgemma-1.5-4b-it	Q1	551 (255)	0.590	0.145	0.973	0.247	0.215
medgemma-1.5-4b-it	Q2	461 (165)	0.672	0.442	0.801	0.492	0.258
medgemma-1.5-4b-it	Q3	424 (38)	0.785	0.447	0.819	0.272	0.188
medgemma-1.5-4b-it	Q4	386 (55)	0.744	0.545	0.776	0.377	0.254
medgemma-1.5-4b-it	Q5	351 (5)	0.866	0.600	0.870	0.113	0.162
medgemma-1.5-4b-it	Q6	334 (7)	0.922	0.571	0.930	0.235	0.263
medgemma-1.5-4b-it	Q7	326 (3)	0.417	0.000	0.421	NA	-0.112
medgemma-1.5-4b-it	Q8	302 (6)	0.940	0.000	0.959	NA	-0.029
medgemma-1.5-4b-it	Q9	261 (26)	0.877	0.038	0.970	0.059	0.015
medgemma-1.5-4b-it	Q10	237 (26)	0.878	0.115	0.972	0.171	0.142
medgemma-1.5-4b-it	Q11	221 (6)	0.955	0.667	0.963	0.444	0.451
medgemma-1.5-4b-it	Q12	218 (0)	0.977	NA	0.977	NA	NA
medgemma-1.5-4b-it	Q13	215 (6)	0.944	0.167	0.967	0.143	0.116
medgemma-1.5-4b-it	Q14	209 (2)	0.967	0.500	0.971	0.222	0.255
medgemma-1.5-4b-it	Q15	195 (10)	0.856	0.100	0.897	0.067	-0.002
medgemma-1.5-4b-it	Overall	4691 (610)	0.793	0.287	0.869	0.265	0.146
medgemma-1.5-4b-it	Subject-level Overall	556 (349)	0.540	0.656	0.343	0.641	-0.001
medgemma-27b-it	Q1	551 (255)	0.760	0.667	0.841	0.720	0.518
medgemma-27b-it	Q2	461 (165)	0.781	0.879	0.726	0.742	0.580
medgemma-27b-it	Q3	424 (38)	0.814	0.711	0.824	0.406	0.366
medgemma-27b-it	Q4	386 (55)	0.728	0.873	0.704	0.478	0.416
medgemma-27b-it	Q5	351 (5)	0.929	1.000	0.928	0.286	0.393
medgemma-27b-it	Q6	334 (7)	0.982	0.429	0.994	0.500	0.498
medgemma-27b-it	Q7	326 (3)	0.960	0.667	0.963	0.235	0.297
medgemma-27b-it	Q8	302 (6)	0.950	0.167	0.966	0.118	0.099
medgemma-27b-it	Q9	261 (26)	0.904	1.000	0.894	0.675	0.675
medgemma-27b-it	Q10	237 (26)	0.911	0.731	0.934	0.644	0.600
medgemma-27b-it	Q11	221 (6)	0.973	0.833	0.977	0.625	0.633
medgemma-27b-it	Q12	218 (0)	0.986	NA	0.986	NA	NA
medgemma-27b-it	Q13	215 (6)	0.930	0.833	0.933	0.400	0.445
medgemma-27b-it	Q14	209 (2)	0.967	0.500	0.971	0.222	0.255
medgemma-27b-it	Q15	195 (10)	0.790	0.600	0.800	0.226	0.213
medgemma-27b-it	Overall	4691 (610)	0.873	0.759	0.890	0.609	0.552
medgemma-27b-it	Subject-level Overall	556 (349)	0.750	0.911	0.478	0.821	0.445

Supplementary Table 5: CHR-P detection performance across the Phi family, stratified by symptom domain. Metrics are computed using a pragmatic PSYCHS-aligned gate derived from predicted severity and frequency. Values are proportions.

Model	Symptom Domain	n (Events)	Accuracy	Sensitivity	Specificity	F1	MCC
Phi-3-medium-4k-instruct	Q1	551 (255)	0.544	0.027	0.990	0.053	0.065
Phi-3-medium-4k-instruct	Q2	461 (165)	0.738	0.539	0.848	0.595	0.409
Phi-3-medium-4k-instruct	Q3	424 (38)	0.870	0.632	0.894	0.466	0.417
Phi-3-medium-4k-instruct	Q4	386 (55)	0.829	0.636	0.861	0.515	0.427
Phi-3-medium-4k-instruct	Q5	351 (5)	0.943	0.400	0.951	0.167	0.184
Phi-3-medium-4k-instruct	Q6	334 (7)	0.979	0.143	0.997	0.222	0.260
Phi-3-medium-4k-instruct	Q7	326 (3)	0.982	0.333	0.988	0.250	0.249
Phi-3-medium-4k-instruct	Q8	302 (6)	0.970	0.000	0.990	NA	-0.014
Phi-3-medium-4k-instruct	Q9	261 (26)	0.927	0.808	0.940	0.689	0.658
Phi-3-medium-4k-instruct	Q10	237 (26)	0.907	0.308	0.981	0.421	0.412
Phi-3-medium-4k-instruct	Q11	221 (6)	0.977	0.667	0.986	0.615	0.606
Phi-3-medium-4k-instruct	Q12	218 (0)	0.991	NA	0.991	NA	NA
Phi-3-medium-4k-instruct	Q13	215 (6)	0.963	0.667	0.971	0.500	0.499
Phi-3-medium-4k-instruct	Q14	209 (2)	0.986	0.000	0.995	NA	-0.007
Phi-3-medium-4k-instruct	Q15	195 (10)	0.851	0.300	0.881	0.171	0.119
Phi-3-medium-4k-instruct	Overall	4691 (610)	0.867	0.326	0.948	0.390	0.326
Phi-3-medium-4k-instruct	Subject-level Overall	556 (349)	0.621	0.550	0.739	0.645	0.282

Supplementary Table 6: Fairness metrics across the Llama family (part 1)

Model	Factor	Reference Category	Demographic Parity	Equalised Odds TPR	Equalised Odds FPR
Llama-3.2-1B-Instruct	Under 18 n=754 (125 events)	Over 18	0.073	0.054	0.058
Llama-3.2-1B-Instruct	Different identity n=71 (17 events)	Male	0.030	-0.141	0.040
Llama-3.2-1B-Instruct	Female n=2176 (259 events)	Male	-0.007	0.003	0.002
Llama-3.2-1B-Instruct	Non-binary n=147 (44 events)	Male	0.054	0.015	-0.006
Llama-3.2-1B-Instruct	American Indian/Alaska Native n=88 (14 events)	White	-0.079	-0.179	-0.071
Llama-3.2-1B-Instruct	Asian n=832 (84 events)	White	-0.032	-0.060	-0.014
Llama-3.2-1B-Instruct	Black or African American n=709 (105 events)	White	-0.020	-0.141	-0.004
Llama-3.2-1B-Instruct	More than one race n=490 (51 events)	White	-0.042	-0.215	-0.008
Llama-3.2-1B-Instruct	Birmingham, UK n=76 (17 events)	Melbourne	0.096	0.082	0.067
Llama-3.2-1B-Instruct	Calgary, Canada n=210 (36 events)	Melbourne	-0.014	-0.068	-0.013
Llama-3.2-1B-Instruct	Cambridge, UK n=92 (14 events)	Melbourne	-0.077	0.090	-0.109
Llama-3.2-1B-Instruct	Georgia, USA n=279 (23 events)	Melbourne	-0.116	-0.146	-0.089
Llama-3.2-1B-Instruct	Hartford, USA n=90 (14 events)	Melbourne	-0.127	-0.267	-0.104
Llama-3.2-1B-Instruct	King's College London, UK n=75 (15 events)	Melbourne	-0.018	0.109	-0.072
Llama-3.2-1B-Instruct	Montreal, Canada n=24 (7 events)	Melbourne	-0.046	-0.338	0.005
Llama-3.2-1B-Instruct	Mt. Sinai, USA n=24 (7 events)	Melbourne	0.079	0.233	-0.053
Llama-3.2-1B-Instruct	Northwell, USA n=279 (28 events)	Melbourne	-0.119	-0.124	-0.101
Llama-3.2-1B-Instruct	Oregon, USA n=123 (12 events)	Melbourne	-0.135	0.209	-0.154
Llama-3.2-1B-Instruct	Penn, USA n=325 (32 events)	Melbourne	-0.147	-0.436	-0.098
Llama-3.2-1B-Instruct	Pittsburgh, USA n=282 (45 events)	Melbourne	-0.153	-0.113	-0.166
Llama-3.2-1B-Instruct	Singapore n=18 (1 events)	Melbourne	-0.227	-0.624	-0.171
Llama-3.2-1B-Instruct	Temple, USA n=94 (4 events)	Melbourne	-0.200	-0.124	-0.166
Llama-3.2-1B-Instruct	UC Irvine, USA n=311 (15 events)	Melbourne	-0.093	0.043	-0.066
Llama-3.2-1B-Instruct	UCSD, USA n=7 (2 events)	Melbourne	-0.052	-0.124	-0.089
Llama-3.2-1B-Instruct	UNC, USA n=797 (138 events)	Melbourne	-0.068	-0.102	-0.072
Llama-3.2-1B-Instruct	WashU, USA n=169 (31 events)	Melbourne	-0.030	0.054	-0.064
Llama-3.2-1B-Instruct	Yale, USA n=673 (60 events)	Melbourne	-0.195	-0.124	-0.181
Llama-3.2-1B-Instruct	Not English n=662 (58 events)	English	-0.031	-0.019	-0.008
Llama-3.2-3B-Instruct	Under 18 n=754 (125 events)	Over 18	0.080	0.015	0.061
Llama-3.2-3B-Instruct	Different identity n=71 (17 events)	Male	0.031	-0.018	-0.026
Llama-3.2-3B-Instruct	Female n=2176 (259 events)	Male	-0.055	-0.013	-0.044
Llama-3.2-3B-Instruct	Non-binary n=147 (44 events)	Male	0.157	0.041	0.078
Llama-3.2-3B-Instruct	American Indian/Alaska Native n=88 (14 events)	White	0.072	0.046	0.060
Llama-3.2-3B-Instruct	Asian n=832 (84 events)	White	-0.032	-0.002	-0.013
Llama-3.2-3B-Instruct	Black or African American n=709 (105 events)	White	0.005	0.017	-0.006
Llama-3.2-3B-Instruct	More than one race n=490 (51 events)	White	-0.046	-0.013	-0.029
Llama-3.2-3B-Instruct	Birmingham, UK n=76 (17 events)	Melbourne	0.054	0.018	0.016
Llama-3.2-3B-Instruct	Calgary, Canada n=210 (36 events)	Melbourne	-0.002	-0.009	-0.015
Llama-3.2-3B-Instruct	Cambridge, UK n=92 (14 events)	Melbourne	-0.118	-0.053	-0.133
Llama-3.2-3B-Instruct	Georgia, USA n=279 (23 events)	Melbourne	-0.184	-0.025	-0.164
Llama-3.2-3B-Instruct	Hartford, USA n=90 (14 events)	Melbourne	-0.108	-0.125	-0.111
Llama-3.2-3B-Instruct	King's College London, UK n=75 (15 events)	Melbourne	-0.004	0.018	-0.042
Llama-3.2-3B-Instruct	Montreal, Canada n=24 (7 events)	Melbourne	0.103	0.018	0.037
Llama-3.2-3B-Instruct	Mt. Sinai, USA n=24 (7 events)	Melbourne	-0.147	0.018	-0.316
Llama-3.2-3B-Instruct	Northwell, USA n=279 (28 events)	Melbourne	-0.180	-0.053	-0.169
Llama-3.2-3B-Instruct	Oregon, USA n=123 (12 events)	Melbourne	-0.271	0.018	-0.276
Llama-3.2-3B-Instruct	Penn, USA n=325 (32 events)	Melbourne	-0.204	-0.107	-0.188
Llama-3.2-3B-Instruct	Pittsburgh, USA n=282 (45 events)	Melbourne	-0.227	0.018	-0.281
Llama-3.2-3B-Instruct	Singapore n=18 (1 events)	Melbourne	-0.342	0.018	-0.316
Llama-3.2-3B-Instruct	Temple, USA n=94 (4 events)	Melbourne	-0.224	0.018	-0.181
Llama-3.2-3B-Instruct	UC Irvine, USA n=311 (15 events)	Melbourne	-0.088	-0.048	-0.039
Llama-3.2-3B-Instruct	UCSD, USA n=7 (2 events)	Melbourne	0.150	0.018	0.108
Llama-3.2-3B-Instruct	UNC, USA n=797 (138 events)	Melbourne	-0.136	-0.040	-0.172
Llama-3.2-3B-Instruct	WashU, USA n=169 (31 events)	Melbourne	0.063	-0.014	0.059
Llama-3.2-3B-Instruct	Yale, USA n=673 (60 events)	Melbourne	-0.262	-0.048	-0.252
Llama-3.2-3B-Instruct	Not English n=662 (58 events)	English	-0.073	0.012	-0.040

Supplementary Table 7: Fairness metrics across the Llama family (part 2)

Model	Factor	Reference Category	Demographic Parity	Equalised Odds TPR	Equalised Odds FPR
Llama-3.1-8B-Instruct	Under 18 n=754 (125 events)	Over 18	0.098	0.077	0.065
Llama-3.1-8B-Instruct	Different identity n=71 (17 events)	Male	0.062	-0.029	0.003
Llama-3.1-8B-Instruct	Female n=2176 (259 events)	Male	-0.020	0.024	-0.005
Llama-3.1-8B-Instruct	Non-binary n=147 (44 events)	Male	0.147	0.011	0.049
Llama-3.1-8B-Instruct	American Indian/Alaska Native n=88 (14 events)	White	-0.016	-0.067	-0.026
Llama-3.1-8B-Instruct	Asian n=832 (84 events)	White	-0.036	0.028	-0.016
Llama-3.1-8B-Instruct	Black or African American n=709 (105 events)	White	0.012	0.004	0.003
Llama-3.1-8B-Instruct	More than one race n=490 (51 events)	White	-0.031	0.030	-0.013
Llama-3.1-8B-Instruct	Birmingham, UK n=76 (17 events)	Melbourne	0.155	0.147	0.093
Llama-3.1-8B-Instruct	Calgary, Canada n=210 (36 events)	Melbourne	0.032	0.063	0.006
Llama-3.1-8B-Instruct	Cambridge, UK n=92 (14 events)	Melbourne	-0.042	0.147	-0.080
Llama-3.1-8B-Instruct	Georgia, USA n=279 (23 events)	Melbourne	-0.099	0.060	-0.067
Llama-3.1-8B-Instruct	Hartford, USA n=90 (14 events)	Melbourne	-0.059	-0.210	-0.038
Llama-3.1-8B-Instruct	King's College London, UK n=75 (15 events)	Melbourne	0.015	0.013	-0.029
Llama-3.1-8B-Instruct	Montreal, Canada n=24 (7 events)	Melbourne	0.083	0.004	-0.019
Llama-3.1-8B-Instruct	Mt. Sinai, USA n=24 (7 events)	Melbourne	0.041	0.004	-0.078
Llama-3.1-8B-Instruct	Northwell, USA n=279 (28 events)	Melbourne	-0.081	0.004	-0.056
Llama-3.1-8B-Instruct	Oregon, USA n=123 (12 events)	Melbourne	-0.113	0.063	-0.096
Llama-3.1-8B-Instruct	Penn, USA n=325 (32 events)	Melbourne	-0.092	-0.228	-0.042
Llama-3.1-8B-Instruct	Pittsburgh, USA n=282 (45 events)	Melbourne	-0.079	0.036	-0.111
Llama-3.1-8B-Instruct	Singapore n=18 (1 events)	Melbourne	-0.181	0.147	-0.137
Llama-3.1-8B-Instruct	Temple, USA n=94 (4 events)	Melbourne	-0.154	-0.353	-0.073
Llama-3.1-8B-Instruct	UC Irvine, USA n=311 (15 events)	Melbourne	-0.077	0.013	-0.013
Llama-3.1-8B-Instruct	UCSD, USA n=7 (2 events)	Melbourne	-0.149	-0.353	-0.196
Llama-3.1-8B-Instruct	UNC, USA n=797 (138 events)	Melbourne	-0.026	0.038	-0.061
Llama-3.1-8B-Instruct	WashU, USA n=169 (31 events)	Melbourne	0.069	0.018	0.051
Llama-3.1-8B-Instruct	Yale, USA n=673 (60 events)	Melbourne	-0.152	-0.020	-0.124
Llama-3.1-8B-Instruct	Not English n=662 (58 events)	English	-0.057	0.055	-0.019
Llama-3.3-70B-Instruct	Under 18 n=754 (125 events)	Over 18	0.092	0.162	0.042
Llama-3.3-70B-Instruct	Different identity n=71 (17 events)	Male	0.083	0.000	0.025
Llama-3.3-70B-Instruct	Female n=2176 (259 events)	Male	-0.023	0.019	-0.009
Llama-3.3-70B-Instruct	Non-binary n=147 (44 events)	Male	0.176	0.099	0.060
Llama-3.3-70B-Instruct	American Indian/Alaska Native n=88 (14 events)	White	0.042	0.084	0.014
Llama-3.3-70B-Instruct	Asian n=832 (84 events)	White	-0.034	0.013	-0.012
Llama-3.3-70B-Instruct	Black or African American n=709 (105 events)	White	0.012	-0.030	0.009
Llama-3.3-70B-Instruct	More than one race n=490 (51 events)	White	0.001	0.051	0.020
Llama-3.3-70B-Instruct	Birmingham, UK n=76 (17 events)	Melbourne	0.194	0.125	0.143
Llama-3.3-70B-Instruct	Calgary, Canada n=210 (36 events)	Melbourne	0.080	0.100	0.055
Llama-3.3-70B-Instruct	Cambridge, UK n=92 (14 events)	Melbourne	0.017	0.041	0.008
Llama-3.3-70B-Instruct	Georgia, USA n=279 (23 events)	Melbourne	-0.068	-0.034	-0.020
Llama-3.3-70B-Instruct	Hartford, USA n=90 (14 events)	Melbourne	-0.067	-0.388	-0.016
Llama-3.3-70B-Instruct	King's College London, UK n=75 (15 events)	Melbourne	0.053	0.117	-0.011
Llama-3.3-70B-Instruct	Montreal, Canada n=24 (7 events)	Melbourne	0.258	0.041	0.199
Llama-3.3-70B-Instruct	Mt. Sinai, USA n=24 (7 events)	Melbourne	0.008	-0.102	-0.095
Llama-3.3-70B-Instruct	Northwell, USA n=279 (28 events)	Melbourne	-0.036	0.005	-0.003
Llama-3.3-70B-Instruct	Oregon, USA n=123 (12 events)	Melbourne	-0.054	0.100	-0.032
Llama-3.3-70B-Instruct	Penn, USA n=325 (32 events)	Melbourne	-0.071	-0.285	-0.009
Llama-3.3-70B-Instruct	Pittsburgh, USA n=282 (45 events)	Melbourne	-0.016	0.028	-0.036
Llama-3.3-70B-Instruct	Singapore n=18 (1 events)	Melbourne	-0.089	0.183	-0.036
Llama-3.3-70B-Instruct	Temple, USA n=94 (4 events)	Melbourne	-0.094	-0.317	-0.006
Llama-3.3-70B-Instruct	UC Irvine, USA n=311 (15 events)	Melbourne	-0.020	0.050	0.051
Llama-3.3-70B-Instruct	UCSD, USA n=7 (2 events)	Melbourne	0.085	0.183	-0.095
Llama-3.3-70B-Instruct	UNC, USA n=797 (138 events)	Melbourne	-0.032	-0.070	-0.048
Llama-3.3-70B-Instruct	WashU, USA n=169 (31 events)	Melbourne	0.060	-0.042	0.050
Llama-3.3-70B-Instruct	Yale, USA n=673 (60 events)	Melbourne	-0.110	-0.167	-0.059
Llama-3.3-70B-Instruct	Not English n=662 (58 events)	English	-0.083	-0.009	-0.042

Supplementary Table 8: Fairness metrics across the Qwen family

Model	Factor	Reference Category	Demographic Parity	Equalised Odds TPR	Equalised Odds FPR
Qwen3-30B-A3B-Instruct-2507	Under 18 n=754 (125 events)	Over 18	0.082	0.068	0.046
Qwen3-30B-A3B-Instruct-2507	Different identity n=71 (17 events)	Male	0.077	-0.041	0.025
Qwen3-30B-A3B-Instruct-2507	Female n=2176 (259 events)	Male	-0.022	0.016	-0.006
Qwen3-30B-A3B-Instruct-2507	Non-binary n=147 (44 events)	Male	0.155	0.044	0.043
Qwen3-30B-A3B-Instruct-2507	American Indian/Alaska Native n=88 (14 events)	White	0.013	-0.002	-0.004
Qwen3-30B-A3B-Instruct-2507	Asian n=832 (84 events)	White	-0.031	0.034	-0.011
Qwen3-30B-A3B-Instruct-2507	Black or African American n=709 (105 events)	White	0.013	0.027	0.000
Qwen3-30B-A3B-Instruct-2507	More than one race n=490 (51 events)	White	-0.006	0.063	0.011
Qwen3-30B-A3B-Instruct-2507	Birmingham, UK n=76 (17 events)	Melbourne	0.172	0.073	0.128
Qwen3-30B-A3B-Instruct-2507	Calgary, Canada n=210 (36 events)	Melbourne	0.018	-0.010	0.001
Qwen3-30B-A3B-Instruct-2507	Cambridge, UK n=92 (14 events)	Melbourne	-0.062	0.002	-0.079
Qwen3-30B-A3B-Instruct-2507	Georgia, USA n=279 (23 events)	Melbourne	-0.129	-0.014	-0.089
Qwen3-30B-A3B-Instruct-2507	Hartford, USA n=90 (14 events)	Melbourne	-0.101	-0.212	-0.089
Qwen3-30B-A3B-Instruct-2507	King's College London, UK n=75 (15 events)	Melbourne	0.019	-0.060	-0.011
Qwen3-30B-A3B-Instruct-2507	Montreal, Canada n=24 (7 events)	Melbourne	0.157	-0.069	0.100
Qwen3-30B-A3B-Instruct-2507	Mt. Sinai, USA n=24 (7 events)	Melbourne	0.032	0.073	-0.135
Qwen3-30B-A3B-Instruct-2507	Northwell, USA n=279 (28 events)	Melbourne	-0.086	-0.105	-0.047
Qwen3-30B-A3B-Instruct-2507	Oregon, USA n=123 (12 events)	Melbourne	-0.147	-0.010	-0.122
Qwen3-30B-A3B-Instruct-2507	Penn, USA n=325 (32 events)	Melbourne	-0.114	-0.302	-0.054
Qwen3-30B-A3B-Instruct-2507	Pittsburgh, USA n=282 (45 events)	Melbourne	-0.071	-0.015	-0.093
Qwen3-30B-A3B-Instruct-2507	Singapore n=18 (1 events)	Melbourne	-0.190	0.073	-0.135
Qwen3-30B-A3B-Instruct-2507	Temple, USA n=94 (4 events)	Melbourne	-0.131	-0.177	-0.050
Qwen3-30B-A3B-Instruct-2507	UC Irvine, USA n=311 (15 events)	Melbourne	-0.038	0.073	0.032
Qwen3-30B-A3B-Instruct-2507	UCSD, USA n=7 (2 events)	Melbourne	0.127	0.073	0.006
Qwen3-30B-A3B-Instruct-2507	UNC, USA n=797 (138 events)	Melbourne	-0.052	-0.064	-0.073
Qwen3-30B-A3B-Instruct-2507	WashU, USA n=169 (31 events)	Melbourne	0.042	0.009	0.016
Qwen3-30B-A3B-Instruct-2507	Yale, USA n=673 (60 events)	Melbourne	-0.178	-0.110	-0.139
Qwen3-30B-A3B-Instruct-2507	Not English n=662 (58 events)	English	-0.063	0.024	-0.022
Qwen3-Next-80B-A3B-Instruct	Under 18 n=754 (125 events)	Over 18	0.093	0.085	0.059
Qwen3-Next-80B-A3B-Instruct	Different identity n=71 (17 events)	Male	0.077	0.041	0.003
Qwen3-Next-80B-A3B-Instruct	Female n=2176 (259 events)	Male	-0.023	-0.006	-0.005
Qwen3-Next-80B-A3B-Instruct	Non-binary n=147 (44 events)	Male	0.157	0.059	0.046
Qwen3-Next-80B-A3B-Instruct	American Indian/Alaska Native n=88 (14 events)	White	0.014	-0.053	0.008
Qwen3-Next-80B-A3B-Instruct	Asian n=832 (84 events)	White	-0.029	0.043	-0.011
Qwen3-Next-80B-A3B-Instruct	Black or African American n=709 (105 events)	White	0.027	-0.005	0.022
Qwen3-Next-80B-A3B-Instruct	More than one race n=490 (51 events)	White	-0.006	0.076	0.009
Qwen3-Next-80B-A3B-Instruct	Birmingham, UK n=76 (17 events)	Melbourne	0.184	0.193	0.114
Qwen3-Next-80B-A3B-Instruct	Calgary, Canada n=210 (36 events)	Melbourne	0.048	-0.002	0.038
Qwen3-Next-80B-A3B-Instruct	Cambridge, UK n=92 (14 events)	Melbourne	-0.039	-0.022	-0.046
Qwen3-Next-80B-A3B-Instruct	Georgia, USA n=279 (23 events)	Melbourne	-0.094	-0.025	-0.053
Qwen3-Next-80B-A3B-Instruct	Hartford, USA n=90 (14 events)	Melbourne	-0.079	-0.307	-0.044
Qwen3-Next-80B-A3B-Instruct	King's College London, UK n=75 (15 events)	Melbourne	0.043	0.059	-0.006
Qwen3-Next-80B-A3B-Instruct	Montreal, Canada n=24 (7 events)	Melbourne	0.235	0.050	0.171
Qwen3-Next-80B-A3B-Instruct	Mt. Sinai, USA n=24 (7 events)	Melbourne	0.027	0.050	-0.123
Qwen3-Next-80B-A3B-Instruct	Northwell, USA n=279 (28 events)	Melbourne	-0.087	-0.129	-0.047
Qwen3-Next-80B-A3B-Instruct	Oregon, USA n=123 (12 events)	Melbourne	-0.077	0.109	-0.060
Qwen3-Next-80B-A3B-Instruct	Penn, USA n=325 (32 events)	Melbourne	-0.076	-0.276	-0.017
Qwen3-Next-80B-A3B-Instruct	Pittsburgh, USA n=282 (45 events)	Melbourne	-0.025	0.037	-0.047
Qwen3-Next-80B-A3B-Instruct	Singapore n=18 (1 events)	Melbourne	-0.112	0.193	-0.064
Qwen3-Next-80B-A3B-Instruct	Temple, USA n=94 (4 events)	Melbourne	-0.106	-0.307	-0.023
Qwen3-Next-80B-A3B-Instruct	UC Irvine, USA n=311 (15 events)	Melbourne	-0.063	0.059	0.002
Qwen3-Next-80B-A3B-Instruct	UCSD, USA n=7 (2 events)	Melbourne	0.062	0.193	-0.123
Qwen3-Next-80B-A3B-Instruct	UNC, USA n=797 (138 events)	Melbourne	-0.040	-0.032	-0.064
Qwen3-Next-80B-A3B-Instruct	WashU, USA n=169 (31 events)	Melbourne	0.090	0.096	0.058
Qwen3-Next-80B-A3B-Instruct	Yale, USA n=673 (60 events)	Melbourne	-0.127	-0.091	-0.087
Qwen3-Next-80B-A3B-Instruct	Not English n=662 (58 events)	English	-0.063	0.106	-0.032

Supplementary Table 9: Fairness metrics across the Gemma family

Model	Factor	Reference Category	Demographic Parity	Equalised Odds TPR	Equalised Odds FPR
gemma-3n-E2B-it	Under 18 n=754 (125 events)	Over 18	0.059	0.063	0.023
gemma-3n-E2B-it	Different identity n=71 (17 events)	Male	0.071	-0.012	0.013
gemma-3n-E2B-it	Female n=2176 (259 events)	Male	-0.040	-0.036	-0.020
gemma-3n-E2B-it	Non-binary n=147 (44 events)	Male	0.149	0.028	0.050
gemma-3n-E2B-it	American Indian/Alaska Native n=88 (14 events)	White	-0.008	-0.114	-0.008
gemma-3n-E2B-it	Asian n=832 (84 events)	White	-0.021	0.017	0.002
gemma-3n-E2B-it	Black or African American n=709 (105 events)	White	0.020	-0.019	0.016
gemma-3n-E2B-it	More than one race n=490 (51 events)	White	-0.026	-0.024	-0.002
gemma-3n-E2B-it	Birmingham, UK n=76 (17 events)	Melbourne	0.092	0.174	0.007
gemma-3n-E2B-it	Calgary, Canada n=210 (36 events)	Melbourne	0.002	0.035	-0.023
gemma-3n-E2B-it	Cambridge, UK n=92 (14 events)	Melbourne	-0.042	0.103	-0.072
gemma-3n-E2B-it	Georgia, USA n=279 (23 events)	Melbourne	-0.113	0.000	-0.080
gemma-3n-E2B-it	Hartford, USA n=90 (14 events)	Melbourne	-0.114	-0.183	-0.108
gemma-3n-E2B-it	King's College London, UK n=75 (15 events)	Melbourne	-0.023	0.041	-0.080
gemma-3n-E2B-it	Montreal, Canada n=24 (7 events)	Melbourne	0.072	0.031	-0.036
gemma-3n-E2B-it	Mt. Sinai, USA n=24 (7 events)	Melbourne	-0.094	-0.254	-0.154
gemma-3n-E2B-it	Northwell, USA n=279 (28 events)	Melbourne	-0.095	0.031	-0.077
gemma-3n-E2B-it	Oregon, USA n=123 (12 events)	Melbourne	-0.140	0.091	-0.132
gemma-3n-E2B-it	Penn, USA n=325 (32 events)	Melbourne	-0.057	0.018	-0.032
gemma-3n-E2B-it	Pittsburgh, USA n=282 (45 events)	Melbourne	-0.065	0.085	-0.103
gemma-3n-E2B-it	Singapore n=18 (1 events)	Melbourne	-0.192	0.174	-0.154
gemma-3n-E2B-it	Temple, USA n=94 (4 events)	Melbourne	-0.175	-0.326	-0.102
gemma-3n-E2B-it	UC Irvine, USA n=311 (15 events)	Melbourne	-0.068	-0.026	-0.007
gemma-3n-E2B-it	UCSD, USA n=7 (2 events)	Melbourne	-0.017	-0.326	-0.013
gemma-3n-E2B-it	UNC, USA n=797 (138 events)	Melbourne	-0.069	-0.043	-0.095
gemma-3n-E2B-it	WashU, USA n=169 (31 events)	Melbourne	0.040	-0.019	0.026
gemma-3n-E2B-it	Yale, USA n=673 (60 events)	Melbourne	-0.169	-0.042	-0.143
gemma-3n-E2B-it	Not English n=662 (58 events)	English	-0.070	0.010	-0.031
gemma-3n-E4B-it	Under 18 n=754 (125 events)	Over 18	0.087	0.103	0.048
gemma-3n-E4B-it	Different identity n=71 (17 events)	Male	0.079	-0.029	0.027
gemma-3n-E4B-it	Female n=2176 (259 events)	Male	-0.023	-0.030	-0.002
gemma-3n-E4B-it	Non-binary n=147 (44 events)	Male	0.171	0.047	0.072
gemma-3n-E4B-it	American Indian/Alaska Native n=88 (14 events)	White	0.000	-0.059	-0.007
gemma-3n-E4B-it	Asian n=832 (84 events)	White	-0.017	0.048	0.001
gemma-3n-E4B-it	Black or African American n=709 (105 events)	White	0.017	0.008	0.009
gemma-3n-E4B-it	More than one race n=490 (51 events)	White	-0.022	-0.008	0.001
gemma-3n-E4B-it	Birmingham, UK n=76 (17 events)	Melbourne	0.195	0.125	0.148
gemma-3n-E4B-it	Calgary, Canada n=210 (36 events)	Melbourne	0.027	0.072	-0.002
gemma-3n-E4B-it	Cambridge, UK n=92 (14 events)	Melbourne	-0.044	0.041	-0.063
gemma-3n-E4B-it	Georgia, USA n=279 (23 events)	Melbourne	-0.107	-0.121	-0.058
gemma-3n-E4B-it	Hartford, USA n=90 (14 events)	Melbourne	-0.062	-0.174	-0.048
gemma-3n-E4B-it	King's College London, UK n=75 (15 events)	Melbourne	0.027	-0.017	-0.007
gemma-3n-E4B-it	Montreal, Canada n=24 (7 events)	Melbourne	0.177	0.041	0.095
gemma-3n-E4B-it	Mt. Sinai, USA n=24 (7 events)	Melbourne	-0.073	-0.245	-0.140
gemma-3n-E4B-it	Northwell, USA n=279 (28 events)	Melbourne	-0.060	0.076	-0.041
gemma-3n-E4B-it	Oregon, USA n=123 (12 events)	Melbourne	-0.093	0.100	-0.077
gemma-3n-E4B-it	Penn, USA n=325 (32 events)	Melbourne	-0.061	-0.223	-0.007
gemma-3n-E4B-it	Pittsburgh, USA n=282 (45 events)	Melbourne	-0.030	0.050	-0.056
gemma-3n-E4B-it	Singapore n=18 (1 events)	Melbourne	-0.128	0.183	-0.082
gemma-3n-E4B-it	Temple, USA n=94 (4 events)	Melbourne	-0.144	-0.317	-0.063
gemma-3n-E4B-it	UC Irvine, USA n=311 (15 events)	Melbourne	-0.063	0.050	0.002
gemma-3n-E4B-it	UCSD, USA n=7 (2 events)	Melbourne	-0.097	-0.317	-0.140
gemma-3n-E4B-it	UNC, USA n=797 (138 events)	Melbourne	-0.035	-0.070	-0.049
gemma-3n-E4B-it	WashU, USA n=169 (31 events)	Melbourne	0.039	-0.075	0.034
gemma-3n-E4B-it	Yale, USA n=673 (60 events)	Melbourne	-0.140	-0.100	-0.101
gemma-3n-E4B-it	Not English n=662 (58 events)	English	-0.061	0.033	-0.024

Supplementary Table 10: Fairness metrics across the MedGemma family

Model	Factor	Reference Category	Demographic Parity	Equalised Odds TPR	Equalised Odds FPR
medgemma-1.5-4b-it	Under 18 n=754 (125 events)	Over 18	0.032	-0.004	0.031
medgemma-1.5-4b-it	Different identity n=71 (17 events)	Male	0.010	0.065	-0.026
medgemma-1.5-4b-it	Female n=2176 (259 events)	Male	-0.015	-0.010	-0.012
medgemma-1.5-4b-it	Non-binary n=147 (44 events)	Male	0.052	0.030	0.028
medgemma-1.5-4b-it	American Indian/Alaska Native n=88 (14 events)	White	-0.008	0.050	-0.024
medgemma-1.5-4b-it	Asian n=832 (84 events)	White	-0.006	-0.021	0.002
medgemma-1.5-4b-it	Black or African American n=709 (105 events)	White	-0.012	-0.097	0.001
medgemma-1.5-4b-it	More than one race n=490 (51 events)	White	-0.023	-0.013	-0.018
medgemma-1.5-4b-it	Birmingham, UK n=76 (17 events)	Melbourne	0.100	0.013	0.107
medgemma-1.5-4b-it	Calgary, Canada n=210 (36 events)	Melbourne	0.015	-0.117	0.037
medgemma-1.5-4b-it	Cambridge, UK n=92 (14 events)	Melbourne	-0.016	0.018	-0.023
medgemma-1.5-4b-it	Georgia, USA n=279 (23 events)	Melbourne	-0.061	0.052	-0.059
medgemma-1.5-4b-it	Hartford, USA n=90 (14 events)	Melbourne	-0.068	-0.054	-0.072
medgemma-1.5-4b-it	King's College London, UK n=75 (15 events)	Melbourne	-0.030	-0.339	0.036
medgemma-1.5-4b-it	Montreal, Canada n=24 (7 events)	Melbourne	0.102	0.232	0.012
medgemma-1.5-4b-it	Mt. Sinai, USA n=24 (7 events)	Melbourne	0.019	0.089	-0.046
medgemma-1.5-4b-it	Northwell, USA n=279 (28 events)	Melbourne	-0.064	-0.054	-0.056
medgemma-1.5-4b-it	Oregon, USA n=123 (12 events)	Melbourne	-0.076	-0.089	-0.065
medgemma-1.5-4b-it	Penn, USA n=325 (32 events)	Melbourne	-0.076	-0.246	-0.048
medgemma-1.5-4b-it	Pittsburgh, USA n=282 (45 events)	Melbourne	-0.076	-0.006	-0.092
medgemma-1.5-4b-it	Singapore n=18 (1 events)	Melbourne	-0.023	0.661	-0.046
medgemma-1.5-4b-it	Temple, USA n=94 (4 events)	Melbourne	-0.094	0.161	-0.086
medgemma-1.5-4b-it	UC Irvine, USA n=311 (15 events)	Melbourne	-0.010	-0.206	0.018
medgemma-1.5-4b-it	UCSD, USA n=7 (2 events)	Melbourne	-0.047	0.161	-0.164
medgemma-1.5-4b-it	UNC, USA n=797 (138 events)	Melbourne	-0.044	-0.064	-0.046
medgemma-1.5-4b-it	WashU, USA n=169 (31 events)	Melbourne	0.059	-0.017	0.068
medgemma-1.5-4b-it	Yale, USA n=673 (60 events)	Melbourne	-0.081	-0.073	-0.071
medgemma-1.5-4b-it	Not English n=662 (58 events)	English	-0.014	-0.072	0.003
medgemma-27b-it	Under 18 n=754 (125 events)	Over 18	0.088	0.082	0.056
medgemma-27b-it	Different identity n=71 (17 events)	Male	0.104	-0.012	0.058
medgemma-27b-it	Female n=2176 (259 events)	Male	-0.019	-0.012	0.000
medgemma-27b-it	Non-binary n=147 (44 events)	Male	0.141	0.042	0.037
medgemma-27b-it	American Indian/Alaska Native n=88 (14 events)	White	-0.014	-0.106	-0.015
medgemma-27b-it	Asian n=832 (84 events)	White	-0.024	0.061	-0.009
medgemma-27b-it	Black or African American n=709 (105 events)	White	0.017	0.023	0.007
medgemma-27b-it	More than one race n=490 (51 events)	White	-0.022	-0.023	0.000
medgemma-27b-it	Birmingham, UK n=76 (17 events)	Melbourne	0.203	0.257	0.128
medgemma-27b-it	Calgary, Canada n=210 (36 events)	Melbourne	0.054	0.035	0.040
medgemma-27b-it	Cambridge, UK n=92 (14 events)	Melbourne	-0.014	0.114	-0.041
medgemma-27b-it	Georgia, USA n=279 (23 events)	Melbourne	-0.077	0.039	-0.046
medgemma-27b-it	Hartford, USA n=90 (14 events)	Melbourne	-0.076	-0.100	-0.078
medgemma-27b-it	King's College London, UK n=75 (15 events)	Melbourne	0.102	0.124	0.056
medgemma-27b-it	Montreal, Canada n=24 (7 events)	Melbourne	0.185	0.114	0.092
medgemma-27b-it	Mt. Sinai, USA n=24 (7 events)	Melbourne	0.019	0.114	-0.144
medgemma-27b-it	Northwell, USA n=279 (28 events)	Melbourne	-0.052	0.007	-0.028
medgemma-27b-it	Oregon, USA n=123 (12 events)	Melbourne	-0.085	0.174	-0.080
medgemma-27b-it	Penn, USA n=325 (32 events)	Melbourne	-0.084	-0.149	-0.045
medgemma-27b-it	Pittsburgh, USA n=282 (45 events)	Melbourne	-0.036	0.124	-0.076
medgemma-27b-it	Singapore n=18 (1 events)	Melbourne	-0.120	0.257	-0.085
medgemma-27b-it	Temple, USA n=94 (4 events)	Melbourne	-0.125	-0.243	-0.055
medgemma-27b-it	UC Irvine, USA n=311 (15 events)	Melbourne	-0.035	0.124	0.019
medgemma-27b-it	UCSD, USA n=7 (2 events)	Melbourne	-0.089	-0.243	-0.144
medgemma-27b-it	UNC, USA n=797 (138 events)	Melbourne	-0.023	-0.004	-0.046
medgemma-27b-it	WashU, USA n=169 (31 events)	Melbourne	0.088	0.128	0.052
medgemma-27b-it	Yale, USA n=673 (60 events)	Melbourne	-0.139	-0.126	-0.103
medgemma-27b-it	Not English n=662 (58 events)	English	-0.067	0.042	-0.033

Supplementary Table 11: Fairness metrics across the Phi family

Model	Factor	Reference Category	Demographic Parity	Equalised Odds TPR	Equalised Odds FPR
Phi-3-medium-4k-instruct	Under 18 n=754 (125 events)	Over 18	0.042	0.020	0.031
Phi-3-medium-4k-instruct	Different identity n=71 (17 events)	Male	0.094	0.159	0.048
Phi-3-medium-4k-instruct	Female n=2176 (259 events)	Male	0.011	0.106	0.005
Phi-3-medium-4k-instruct	Non-binary n=147 (44 events)	Male	0.088	0.065	0.052
Phi-3-medium-4k-instruct	American Indian/Alaska Native n=88 (14 events)	White	-0.038	-0.135	-0.028
Phi-3-medium-4k-instruct	Asian n=832 (84 events)	White	-0.017	-0.004	-0.007
Phi-3-medium-4k-instruct	Black or African American n=709 (105 events)	White	-0.008	-0.026	-0.009
Phi-3-medium-4k-instruct	More than one race n=490 (51 events)	White	-0.026	-0.075	-0.010
Phi-3-medium-4k-instruct	Birmingham, UK n=76 (17 events)	Melbourne	0.079	0.036	0.061
Phi-3-medium-4k-instruct	Calgary, Canada n=210 (36 events)	Melbourne	-0.009	-0.098	0.001
Phi-3-medium-4k-instruct	Cambridge, UK n=92 (14 events)	Melbourne	0.023	0.267	-0.023
Phi-3-medium-4k-instruct	Georgia, USA n=279 (23 events)	Melbourne	-0.050	-0.028	-0.031
Phi-3-medium-4k-instruct	Hartford, USA n=90 (14 events)	Melbourne	-0.041	-0.019	-0.048
Phi-3-medium-4k-instruct	King's College London, UK n=75 (15 events)	Melbourne	0.002	0.024	-0.024
Phi-3-medium-4k-instruct	Montreal, Canada n=24 (7 events)	Melbourne	0.132	0.052	0.102
Phi-3-medium-4k-instruct	Mt. Sinai, USA n=24 (7 events)	Melbourne	-0.035	-0.090	-0.074
Phi-3-medium-4k-instruct	Northwell, USA n=279 (28 events)	Melbourne	-0.065	-0.162	-0.038
Phi-3-medium-4k-instruct	Oregon, USA n=123 (12 events)	Melbourne	-0.021	0.124	-0.020
Phi-3-medium-4k-instruct	Penn, USA n=325 (32 events)	Melbourne	-0.057	-0.220	-0.023
Phi-3-medium-4k-instruct	Pittsburgh, USA n=282 (45 events)	Melbourne	-0.030	0.024	-0.045
Phi-3-medium-4k-instruct	Singapore n=18 (1 events)	Melbourne	-0.118	-0.376	-0.074
Phi-3-medium-4k-instruct	Temple, USA n=94 (4 events)	Melbourne	-0.055	-0.376	-0.007
Phi-3-medium-4k-instruct	UC Irvine, USA n=311 (15 events)	Melbourne	-0.022	0.024	0.007
Phi-3-medium-4k-instruct	UCSD, USA n=7 (2 events)	Melbourne	0.024	0.124	-0.074
Phi-3-medium-4k-instruct	UNC, USA n=797 (138 events)	Melbourne	-0.038	-0.101	-0.035
Phi-3-medium-4k-instruct	WashU, USA n=169 (31 events)	Melbourne	0.035	-0.021	0.035
Phi-3-medium-4k-instruct	Yale, USA n=673 (60 events)	Melbourne	-0.074	-0.093	-0.053
Phi-3-medium-4k-instruct	Not English n=662 (58 events)	English	-0.042	-0.031	-0.024

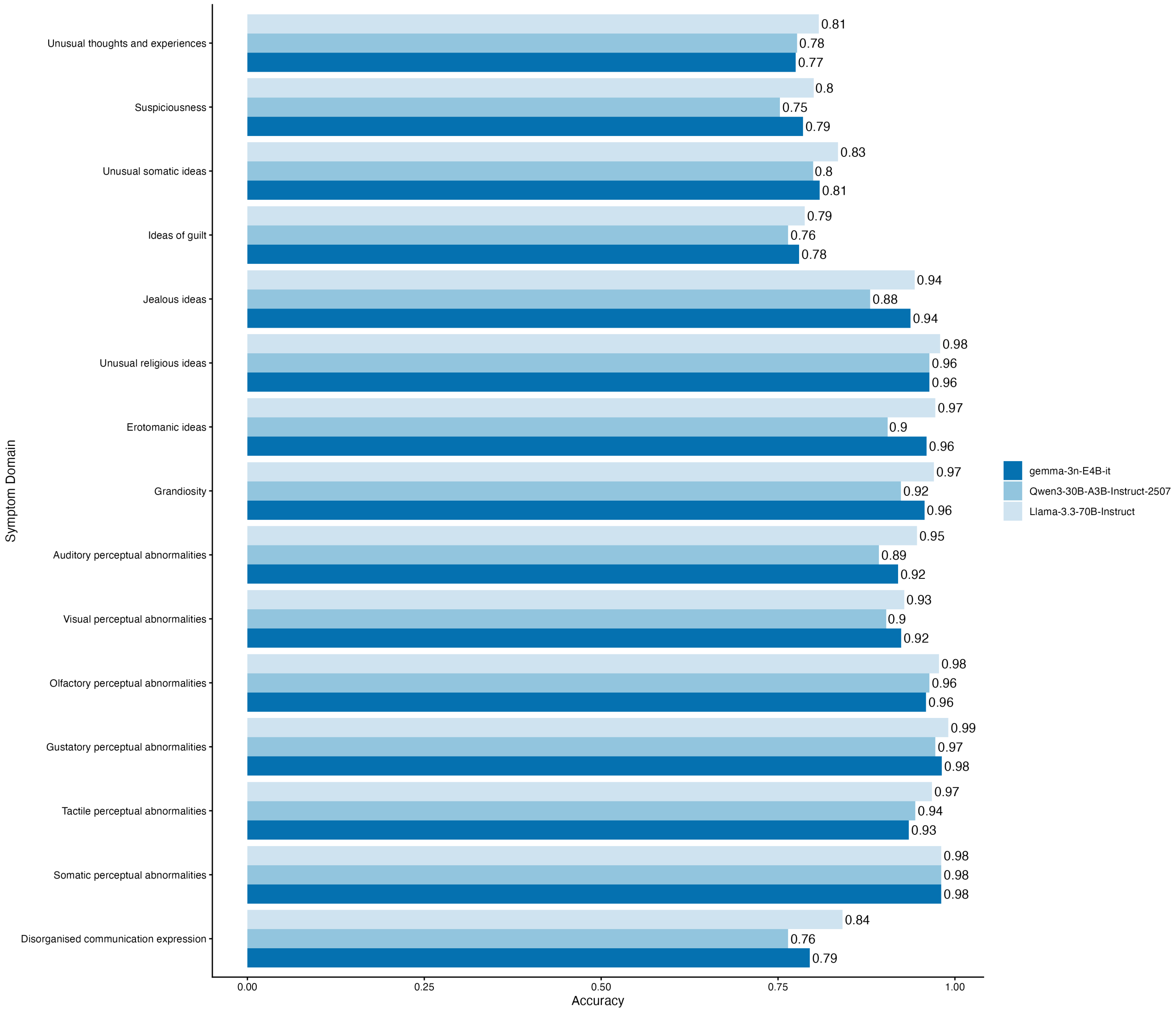

Supplementary Figure 4: Accuracy stratified by symptom domain

Prompt templates for PSYCHS ratings

We used the following instruction CoT templates to prompt the LLM to rate the severity/intensity and frequency of the PSYCHS using evidence contained in each interview transcript. They were used for the full set of 15 PSYCHS-aligned domains: (i) unusual thoughts and experiences; (ii) suspiciousness; (iii) unusual somatic ideas; (iv) ideas of guilt; (v) jealous ideas; (vi) unusual religious ideas; (vii) erotomanic ideas; (viii) grandiosity; (ix) auditory perceptual abnormalities; (x) visual perceptual abnormalities; (xi) olfactory perceptual abnormalities; (xii) gustatory perceptual abnormalities; (xiii) tactile perceptual abnormalities; (xiv) somatic perceptual abnormalities; and (xv) disorganized communication/expression.

Unusual thoughts and experiences

```
You are a clinician specialised in the clinical high risk for psychosis state.  Your task is to rate the overall
severity and frequency of the Unusual Thoughts and Experiences subscale of the PSYCHS according to an interview
transcript.

---
### Transcript:
{transcript}
---

### Rating criteria

**Severity (0-6):**
0 (Absent) = No unusual thought content.
1 (Questionable) = Common ‘‘mind tricks’’ (e.g., déjà vu); quickly dismissed; no meaningful distress/interference.
2 (Mild) = Unusual ideas/experiences within cultural norms (e.g., superstitions, premonitions, fantasy preoccupation);
minimal distress; minimal interference; scepticism easily generated.
3 (Moderate) = Meaningful/puzzling/unwilled experiences that are not easily ignored; person tends to view them
as probably imaginary; some distress or impact; doubt generated with little effort.
4 (Marked) = Unlikely/referential ideas with a sense they may be real; distress and/or interference is present;
doubt possible but requires effort.
5 (Severe but not psychotic) = Peculiar/improbable ideas that seem real; distress/interference clearly present;
doubt mainly only when challenged.
6 (Psychotic and very severe) = Strange/highly improbable ideas felt completely real; delusional conviction; severe
distress and/or marked behavioural/functional interference.

**Frequency (0-6):**
0 = Absent
1 = Less than one day a month
2 = One day a month to two days a week - < 1 hour/day
3 = One day a month to two days a week - > 1 hour/day OR 3--6 days/week - < 1 hour/day
4 = 3--6 days/week - > 1 hour/day OR daily - < 1 hour/day
5 = Daily - > 1 hour/day OR several times/day
6 = Continuous

---

### Output format
Return your response only as a single valid JSON, matching this exact schema:


{
  "severity": <integer 0-6>,
  "frequency": <integer 0-6>,
  "summary": "<1 paragraph (3-5 sentences) summarizing symptoms
  alongside the severity and frequency for a psychiatrist’s initial assessment>"
}
```

Suspiciousness

```
You are a clinician specialised in the clinical high risk for psychosis state.  Your task is to rate the overall
severity and frequency of the Suspiciousness/paranoia (including persecutory ideas of reference) subscale of the
PSYCHS according to an interview transcript.

---
### Transcript:
{transcript}
---

### Rating criteria
```

Severity (0-6):
0 (Absent) = No suspicious/paranoid ideas.
1 (Questionable) = Cautiousness or uncertainty about others' intent that is plausibly reality-based; readily dismissed; minimal distress/interference.
2 (Mild) = Suspicion beyond average but still within cultural norms (e.g., increased self-consciousness, worry about scrutiny); mild distress; little interference; scepticism easy.
3 (Moderate) = Plausible but likely-imaginary suspicions (e.g., others thinking/saying negative things, untrustworthiness); some distress/impact; doubt with little effort.
4 (Marked) = Ideas arise without logical evidence and feel possibly real (e.g., being targeted by negative attention; others may wish harm); distress/interference present; doubt only with effort.
5 (Severe but not psychotic) = Improbable suspicions that seem real despite lack of evidence; clear distress/interference; doubt mainly when challenged.
6 (Psychotic and very severe) = Highly improbable persecutory beliefs felt completely real despite contrary evidence; delusional conviction; severe distress and/or marked interference/behavioural impact.

Frequency (0-6):
0 = Absent
1 = Less than one day a month
2 = One day a month to two days a week - $<$ 1 hour/day
3 = One day a month to two days a week - $>$ 1 hour/day OR 3--6 days/week - $<$ 1 hour/day
4 = 3--6 days/week - $>$ 1 hour/day OR daily - $<$ 1 hour/day
5 = Daily - $>$ 1 hour/day OR several times/day
6 = Continuous

Output format
Return your response only as a single valid JSON:

```
{
  "severity": <integer 0-6>,
  "frequency": <integer 0-6>,
  "summary": "<1 paragraph (3-5 sentences) summarizing symptoms
  alongside the severity and frequency for a psychiatrist's initial assessment>"
}
```

Unusual somatic ideas

You are a clinician specialised in the clinical high risk for psychosis state. Your task is to rate the overall severity and frequency of the Unusual Somatic Ideas subscale of the PSYCHS according to an interview transcript.

Transcript:
{transcript}

Rating criteria

Severity (0-6):
0 (Absent) = No unusual somatic ideas.
1 (Questionable) = Possibly reality-based over-focus on body/body-part traits; easily dismissed; minimal distress/interference.
2 (Mild) = Concerns about body/body-part traits beyond average but within cultural norms; mild distress; little interference; scepticism easy.
3 (Moderate) = Plausible/meaningful preoccupation with body traits (may cite ''evidence'') but person views as probably imaginary; some distress/impact; doubt with little effort.
4 (Marked) = Exaggerated somatic ideas arising without logical evidence and feel possibly real; distress/interference present; doubt only with effort.
5 (Severe but not psychotic) = Improbable somatic beliefs that seem real despite lack of evidence; clear distress/interference; doubt mainly when challenged.
6 (Psychotic and very severe) = Highly improbable somatic beliefs felt completely real despite contrary evidence; delusional conviction; severe distress and/or marked interference/behaviour change.

Frequency (0-6):
0 = Absent
1 = Less than one day a month
2 = One day a month to two days a week - $<$ 1 hour/day
3 = One day a month to two days a week - $>$ 1 hour/day OR 3--6 days/week - $<$ 1 hour/day
4 = 3--6 days/week - $>$ 1 hour/day OR daily - $<$ 1 hour/day
5 = Daily - $>$ 1 hour/day OR several times/day
6 = Continuous

```
### Output format
Return only JSON:

{
  "severity": <integer 0-6>,
  "frequency": <integer 0-6>,
  "summary": "<1 paragraph (3-5 sentences) summarizing symptoms
  alongside the severity and frequency for a psychiatrist’s initial assessment>"
}
```

Ideas of guilt

```
You are a clinician specialised in the clinical high risk for psychosis state.  Your task is to rate the overall
severity and frequency of the Ideas of Guilt subscale of the PSYCHS according to an interview transcript.

---
### Transcript:
{transcript}
---

### Rating criteria

**Severity (0-6):**
0 (Absent) = No excessive/unusual guilt ideas.
1 (Questionable) = Reality-based uncertainty about impact of actions; readily corrected; minimal distress/interference.
2 (Mild) = Overly remorseful within cultural norms; mild distress; little interference; scepticism easy.
3 (Moderate) = Self-blame that is meaningful/plausible (may cite ‘‘evidence’’) but likely imaginary/exaggerated;
some distress/impact; doubt with little effort.
4 (Marked) = Excessive self-blame arising without logical evidence; feels possibly real; distress/interference
present; doubt only with effort.
5 (Severe but not psychotic) = Improbable guilt/responsibility (incl.  events outside control) that seems real;
clear distress/interference; doubt mainly when challenged.
6 (Psychotic and very severe) = Highly improbable guilt/responsibility felt completely real despite contrary evidence;
delusional conviction; severe distress and/or marked functional/behavioural interference.

**Frequency (0-6):**
0 = Absent
1 = Less than one day a month
2 = One day a month to two days a week - < 1 hour/day
3 = One day a month to two days a week - > 1 hour/day OR 3--6 days/week - < 1 hour/day
4 = 3--6 days/week - > 1 hour/day OR daily - < 1 hour/day
5 = Daily - > 1 hour/day OR several times/day
6 = Continuous

---

### Output format
Return only JSON:

{
  "severity": <integer 0-6>,
  "frequency": <integer 0-6>,
  "summary": "<1 paragraph (3-5 sentences) summarizing symptoms
  alongside the severity and frequency for a psychiatrist’s initial assessment>"
}
```

Jealous ideas

```
You are a clinician specialised in the clinical high risk for psychosis state.  Your task is to rate the overall
severity and frequency of the Jealous Ideas subscale of the PSYCHS according to an interview transcript.

---
### Transcript:
{transcript}
---

### Rating criteria

**Severity (0-6):**
0 (Absent) = No jealous ideas.
1 (Questionable) = Reality-based uncertainty about others’ allegiance; easily dismissed; minimal distress/interference.
2 (Mild) = Envy/jealous thoughts within cultural norms and easily dismissed; mild distress; little interference.
```

```
3 (Moderate) = Plausible jealousy concerns (e.g., worries about infidelity) that feel meaningful but are likely
imaginary; some distress/impact; doubt with little effort.
4 (Marked) = Jealous ideas arise without logical evidence and feel possibly real (e.g., suspected infidelity);
distress/interference present; doubt only with effort.
5 (Severe but not psychotic) = Improbable jealousy beliefs that seem real despite lack of evidence;
clear distress/interference; doubt mainly when challenged.
6 (Psychotic and very severe) = Highly improbable jealousy beliefs felt completely real despite contrary evidence;
delusional conviction; severe distress (e.g., intense anger) and/or marked interference/behavioural change.

**Frequency (0-6):**
0 = Absent
1 = Less than one day a month
2 = One day a month to two days a week - < 1 hour/day
3 = One day a month to two days a week - > 1 hour/day OR 3--6 days/week - < 1 hour/day
4 = 3--6 days/week - > 1 hour/day OR daily - < 1 hour/day
5 = Daily - > 1 hour/day OR several times/day
6 = Continuous

---

### Output format
Return only JSON:


{
  "severity": <integer 0-6>,
  "frequency": <integer 0-6>,
  "summary": "<1 paragraph (3-5 sentences) summarizing symptoms
  alongside the severity and frequency for a psychiatrist's initial assessment>"
}
```

Unusual religious ideas

```
You are a clinician specialised in the clinical high risk for psychosis state.  Your task is to rate the overall
severity and frequency of the Unusual Religious Ideas subscale of the PSYCHS according to an interview transcript.

---
### Transcript:
{transcript}
---

### Rating criteria

**Severity (0-6):**
0 (Absent) = No unusual religious/spiritual ideas.
1 (Questionable) = Slightly unusual but close to commonly held beliefs; easily reconsidered; minimal distress/interference.
2 (Mild) = Religious/spiritual ideas beyond average but still within cultural norms; mild distress/impact; scepticism
easy.
3 (Moderate) = Somewhat idiosyncratic and somewhat discordant from cultural norms; meaningful experiences; some
distress/impact; doubt with little effort.
4 (Marked) = Clearly idiosyncratic and clearly discordant from cultural norms; distress/interference present;
doubt only with effort.
5 (Severe but not psychotic) = Particularly idiosyncratic/discordant beliefs that seem real; clear distress/interference;
doubt mainly when challenged.
6 (Psychotic and very severe) = Extremely idiosyncratic/discordant beliefs held with delusional conviction; severe
distress and/or marked interference/behavioural impact.

**Frequency (0-6):**
0 = Absent
1 = Less than one day a month
2 = One day a month to two days a week - < 1 hour/day
3 = One day a month to two days a week - > 1 hour/day OR 3--6 days/week - < 1 hour/day
4 = 3--6 days/week - > 1 hour/day OR daily - < 1 hour/day
5 = Daily - > 1 hour/day OR several times/day
6 = Continuous

---

### Output format
Return only JSON:


{
  "severity": <integer 0-6>,
  "frequency": <integer 0-6>,
```

```
  "summary": "<1 paragraph (3-5 sentences) summarizing symptoms
  alongside the severity and frequency for a psychiatrist's initial assessment>"
}
```

Erotomanic ideas

```
You are a clinician specialised in the clinical high risk for psychosis state.  Your task is to rate the overall
severity and frequency of the Erotomanic Ideas subscale of the PSYCHS according to an interview transcript.

---
### Transcript:
{transcript}
---

### Rating criteria

**Severity (0-6):**
0 (Absent) = No erotomanic ideas.
1 (Questionable) = Misreads friendliness as flirtation in a reality-based way; quickly dismisses;
minimal distress/interference.
2 (Mild) = Crush/attributing affection within cultural norms; mild distress/impact; scepticism easy.
3 (Moderate) = Plausible but likely-imaginary belief that someone is romantically interested; meaningful/preoccupying;
some distress/impact; doubt with little effort.
4 (Marked) = Ideas arise without logical evidence and feel possibly real (e.g., suspected love/adoration);
distress/interference present; doubt only with effort.
5 (Severe but not psychotic) = Improbable erotomanic belief that seems real despite lack of evidence;
clear distress/interference; doubt mainly when challenged.
6 (Psychotic and very severe) = Highly improbable erotomanic belief felt completely real despite clear contrary
evidence; delusional conviction; severe distress and/or marked interference/behavioural impact.

**Frequency (0-6):**
0 = Absent
1 = Less than one day a month
2 = One day a month to two days a week - < 1 hour/day
3 = One day a month to two days a week - > 1 hour/day OR 3--6 days/week - < 1 hour/day
4 = 3--6 days/week - > 1 hour/day OR daily - < 1 hour/day
5 = Daily - > 1 hour/day OR several times/day
6 = Continuous

---

### Output format
Return only JSON:

{
  "severity": <integer 0-6>,
  "frequency": <integer 0-6>,
  "summary": "<1 paragraph (3-5 sentences) summarizing symptoms
  alongside the severity and frequency for a psychiatrist's initial assessment>"
}
```

Grandiosity

```
You are a clinician specialised in the clinical high risk for psychosis state.  Your task is to rate the overall
severity and frequency of the Grandiosity subscale of the PSYCHS according to an interview transcript.

---
### Transcript:
{transcript}
---

### Rating criteria

**Severity (0-6):**
0 (Absent) = No grandiosity.
1 (Questionable) = Private thoughts of being better than others; easily corrected; no meaningful interference.
2 (Mild) = Culturally plausible or mostly private beliefs of special aptitude; mild impact; scepticism easy.
3 (Moderate) = Plausible notions of being unusually gifted and/or boastful speech; some functional/social impact;
doubt with little effort.
4 (Marked) = Grandiose beliefs arise without logical evidence and feel possibly real (e.g., special influence/abilities);
interference present; doubt only with effort.
5 (Severe but not psychotic) = Improbable beliefs of superior intellect/power/fame that seem real; clear interference
(plans/behaviour affected); doubt mainly when challenged.
```

```
6 (Psychotic and very severe) = Highly improbable grandiose beliefs felt completely real despite contrary evidence;
delusional conviction; marked interference and/or clear behavioural enactment.

**Frequency (0-6):**
0 = Absent
1 = Less than one day a month
2 = One day a month to two days a week - < 1 hour/day
3 = One day a month to two days a week - > 1 hour/day OR 3--6 days/week - < 1 hour/day
4 = 3--6 days/week - > 1 hour/day OR daily - < 1 hour/day
5 = Daily - > 1 hour/day OR several times/day
6 = Continuous

---

### Output format
Return only JSON:

{
  "severity": <integer 0-6>,
  "frequency": <integer 0-6>,
  "summary": "<1 paragraph (3-5 sentences) summarizing symptoms
  alongside the severity and frequency for a psychiatrist’s initial assessment>"
}
```

Auditory perceptual abnormalities

```
You are a clinician specialised in the clinical high risk for psychosis state.  Your task is to rate the overall
severity and frequency of Auditory Perceptual Abnormalities (including source/insight, distress, and interference)
in the PSYCHS according to an interview transcript.

---
### Transcript:
{transcript}
---

### Rating criteria

**Severity (0-6):**
0 (Absent) = No unusual auditory perceptual experiences.
1 (Questionable) = Increased attention to sounds or brief misidentification of common sounds; recognised as ordinary;
minimal distress/interference.
2 (Mild) = Sensitivity changes, hypnagogic/hypnopompic phenomena, slight auditory illusions; insight largely intact;
mild distress; little interference.
3 (Moderate) = No-stimulus experiences without discernible words (e.g., indistinct murmuring) or clear distortions
of real sounds; person tends to think ‘‘probably not real’’; some distress/impact.
4 (Marked) = No-stimulus experiences with some discernible words (e.g., name called) but not complex; source may
feel possibly real; distress/interference present.
5 (Severe but not psychotic) = Fully discernible words/sentences but lacks full quality of true perception OR
loud internal thoughts mostly perceived as a voice; source seems real and mostly distinct; clear distress/interference.
6 (Psychotic and very severe) = Vivid, true-perception-quality voices/sounds (inside or outside) held as completely
real/distinct; severe distress and/or marked interference/behavioural impact.

**Frequency (0-6):**
0 = Absent
1 = Less than one day a month
2 = One day a month to two days a week - < 1 hour/day
3 = One day a month to two days a week - > 1 hour/day OR 3--6 days/week - < 1 hour/day
4 = 3--6 days/week - > 1 hour/day OR daily - < 1 hour/day
5 = Daily - > 1 hour/day OR several times/day
6 = Continuous

---

### Output format
Return only JSON:

{
  "severity": <integer 0-6>,
  "frequency": <integer 0-6>,
  "summary": "<1 paragraph (3-5 sentences) summarizing symptoms
  alongside the severity and frequency for a psychiatrist’s initial assessment>"
}
```

Visual perceptual abnormalities

```
You are a clinician specialised in the clinical high risk for psychosis state.  Your task is to rate the overall
severity and frequency of Visual Perceptual Abnormalities (including source/insight, distress, and interference)
in the PSYCHS according to an interview transcript.

---
### Transcript:
{transcript}
---

### Rating criteria

**Severity (0-6):**
0 (Absent) = No unusual visual perceptual experiences.
1 (Questionable) = Momentary misidentification in peripheral vision or increased attention to ordinary visual
phenomena; recognised as ordinary; minimal distress/interference.
2 (Mild) = Shadows/sensitivity changes, hypnagogic/hypnopompic phenomena, slight illusions within cultural norms;
insight largely intact; mild distress; little interference.
3 (Moderate) = No-stimulus flashes/movement/undefined shapes OR unusual distortions significantly different from
stimulus; person tends to think ‘‘probably not real’’; some distress/impact.
4 (Marked) = No-stimulus experiences with some identifiable features (ill-defined but recognisable figures/objects);
source may feel possibly real; distress/interference present.
5 (Severe but not psychotic) = Fully discernible forms but lacks true-perception quality (can describe how it
differs from real); source seems real and mostly distinct; clear distress/interference.
6 (Psychotic and very severe) = Vivid, true-perception-quality visions (exactly like real people/creatures/objects);
experienced as completely real/distinct; severe distress and/or marked interference.

**Frequency (0-6):**
0 = Absent
1 = Less than one day a month
2 = One day a month to two days a week - < 1 hour/day
3 = One day a month to two days a week - > 1 hour/day OR 3--6 days/week - < 1 hour/day
4 = 3--6 days/week - > 1 hour/day OR daily - < 1 hour/day
5 = Daily - > 1 hour/day OR several times/day
6 = Continuous

---

### Output format
Return only JSON:


{
  "severity": <integer 0-6>,
  "frequency": <integer 0-6>,
  "summary": "<1 paragraph (3-5 sentences) summarizing symptoms
  alongside the severity and frequency for a psychiatrist’s initial assessment>"
}
```

Olfactory perceptual abnormalities

```
You are a clinician specialised in the clinical high risk for psychosis state.  Your task is to rate the overall
severity and frequency of Olfactory Perceptual Abnormalities (including source/insight, distress, and interference)
in the PSYCHS according to an interview transcript.

---
### Transcript:
{transcript}
---

### Rating criteria

**Severity (0-6):**
0 (Absent) = No unusual olfactory experiences.
1 (Questionable) = Ordinary smells given extra attention; recognised as ordinary; minimal distress/interference.
2 (Mild) = Odour sensitivity changes, hypnagogic/hypnopompic odours, culturally plausible experiences; insight
largely intact; mild distress; little interference.
3 (Moderate) = No-stimulus vague odours (e.g., non-specific sweet smell) OR unusual odour illusions; person tends
to think ‘‘probably not real’’; some distress/impact.
4 (Marked) = No-stimulus identifiable odours (e.g., sea air/salt water) felt possibly real; distress/interference
present.
5 (Severe but not psychotic) = Detailed odours without true-perception quality (can describe differences); source
seems real/mostly distinct; clear distress/interference.
```

```
6 (Psychotic and very severe) = Vivid, true-perception-quality smells (e.g., rotting flesh clinging) experienced
as completely real/distinct; severe distress and/or marked interference.

**Frequency (0-6):**
0 = Absent
1 = Less than one day a month
2 = One day a month to two days a week - < 1 hour/day
3 = One day a month to two days a week - > 1 hour/day OR 3--6 days/week - < 1 hour/day
4 = 3--6 days/week - > 1 hour/day OR daily - < 1 hour/day
5 = Daily - > 1 hour/day OR several times/day
6 = Continuous

---

### Output format
Return only JSON:


{
  "severity": <integer 0-6>,
  "frequency": <integer 0-6>,
  "summary": "<1 paragraph (3-5 sentences) summarizing symptoms
  alongside the severity and frequency for a psychiatrist's initial assessment>"
}
```

Gustatory perceptual abnormalities

```
You are a clinician specialised in the clinical high risk for psychosis state.  Your task is to rate the overall
severity and frequency of Gustatory Perceptual Abnormalities (including source/insight, distress, and interference)
in the PSYCHS according to an interview transcript.

---
### Transcript:
{transcript}
---

### Rating criteria

**Severity (0-6):**
0 (Absent) = No unusual gustatory experiences.
1 (Questionable) = Ordinary tastes given extra attention; recognised as ordinary; minimal distress/interference.
2 (Mild) = Taste sensitivity changes, hypnagogic/hypnopompic tastes, culturally plausible experiences; insight
largely intact; mild distress; little interference.
3 (Moderate) = No-stimulus vague tastes (non-specific sweet/sour) OR unusual taste illusions (e.g., water tastes
tainted); person tends to think ‘‘probably not real’’; some distress/impact.
4 (Marked) = No-stimulus identifiable tastes (e.g., metallic taste) felt possibly real; distress/interference
present.
5 (Severe but not psychotic) = Detailed tastes without true-perception quality (e.g., taste like blood/spoiled
food) experienced as seeming real/mostly distinct; clear distress/interference.
6 (Psychotic and very severe) = Vivid, true-perception-quality tastes (e.g., rotten flesh/faeces) experienced
as completely real/distinct; severe distress and/or marked interference.

**Frequency (0-6):**
0 = Absent
1 = Less than one day a month
2 = One day a month to two days a week - < 1 hour/day
3 = One day a month to two days a week - > 1 hour/day OR 3--6 days/week - < 1 hour/day
4 = 3--6 days/week - > 1 hour/day OR daily - < 1 hour/day
5 = Daily - > 1 hour/day OR several times/day
6 = Continuous

---

### Output format
Return only JSON:


{
  "severity": <integer 0-6>,
  "frequency": <integer 0-6>,
  "summary": "<1 paragraph (3-5 sentences) summarizing symptoms
  alongside the severity and frequency for a psychiatrist's initial assessment>"
}
```

Tactile perceptual abnormalities

You are a clinician specialised in the clinical high risk for psychosis state. Your task is to rate the overall severity and frequency of Tactile Perceptual Abnormalities (including source/insight, distress, and interference) in the PSYCHS according to an interview transcript.

Transcript:
{transcript}

Rating criteria

Severity (0-6):
0 (Absent) = No unusual tactile experiences.
1 (Questionable) = Ordinary tactile sensations given extra attention; recognised as ordinary; minimal distress/interference.
2 (Mild) = Sensory changes (air current, shiver), hypnagogic/hypnopompic sensations, culturally plausible experiences; insight largely intact; mild distress; little interference.
3 (Moderate) = No-stimulus vague sensations (tingling/prickling/warmth/cold) OR unusual tactile illusions; person tends to think ‘‘probably not real’’; some distress/impact.
4 (Marked) = No-stimulus identifiable sensations (pinpricks, stroking hair, touching body part) felt possibly real; distress/interference present.
5 (Severe but not psychotic) = Detailed sensations without full true-perception quality (bugs crawling, gripping, needles); source seems real/mostly distinct; clear distress/interference.
6 (Psychotic and very severe) = Vivid, true-perception-quality tactile experiences (e.g., sexual touch; extreme bodily sensations) experienced as completely real/distinct; severe distress and/or marked interference.

Frequency (0-6):
0 = Absent
1 = Less than one day a month
2 = One day a month to two days a week - $<$ 1 hour/day
3 = One day a month to two days a week - $>$ 1 hour/day OR 3--6 days/week - $<$ 1 hour/day
4 = 3--6 days/week - $>$ 1 hour/day OR daily - $<$ 1 hour/day
5 = Daily - $>$ 1 hour/day OR several times/day
6 = Continuous

Output format
Return only JSON:

```
{
  "severity": <integer 0-6>,
  "frequency": <integer 0-6>,
  "summary": "<1 paragraph (3-5 sentences) summarizing symptoms
  alongside the severity and frequency for a psychiatrist’s initial assessment>"
}
```

Somatic perceptual abnormalities

You are a clinician specialised in the clinical high risk for psychosis state. Your task is to rate the overall severity and frequency of Somatic Perceptual Abnormalities (including source/insight, distress, and interference) in the PSYCHS according to an interview transcript.

Transcript:
{transcript}

Rating criteria

Severity (0-6):
0 (Absent) = No unusual somatic perceptual experiences.
1 (Questionable) = Ordinary internal sensations given extra attention (e.g., bloatedness); recognised as ordinary; minimal distress/interference.
2 (Mild) = Culturally plausible internal sensations (e.g., feeling heat inside), hypnagogic/hypnopompic somatic sensations; insight largely intact; mild distress; little interference.
3 (Moderate) = No-stimulus vague internal sensations (organs swollen/itchy; blood coursing) OR unusual somatic illusions; person tends to think ‘‘probably not real’’; some distress/impact.
4 (Marked) = No-stimulus identifiable internal sensations (organs moving/distorted; electricity inside) felt possibly real; distress/interference present.
5 (Severe but not psychotic) = Detailed internal sensations without true-perception quality (touched inside; organs diseased/altered) experienced as seeming real/mostly distinct; clear distress/interference.

```
6 (Psychotic and very severe) = Vivid, true-perception-quality extreme internal experiences (e.g., animals/aliens
inside) experienced as completely real/distinct; severe distress and/or marked interference.

**Frequency (0-6):**
0 = Absent
1 = Less than one day a month
2 = One day a month to two days a week - < 1 hour/day
3 = One day a month to two days a week - > 1 hour/day OR 3--6 days/week - < 1 hour/day
4 = 3--6 days/week - > 1 hour/day OR daily - < 1 hour/day
5 = Daily - > 1 hour/day OR several times/day
6 = Continuous

---

### Output format
Return only JSON:

{
  "severity": <integer 0-6>,
  "frequency": <integer 0-6>,
  "summary": "<1 paragraph (3-5 sentences) summarizing symptoms
  alongside the severity and frequency for a psychiatrist's initial assessment>"
}
```

Disorganised communication expression

```
You are a clinician specialised in the clinical high risk for psychosis state. Your task is to rate the overall
severity and frequency of Disorganised Communication Expression (including self-correction, distress, and interference)
in the PSYCHS according to an interview transcript.

---
### Transcript:
{transcript}
---

### Rating criteria

**Severity (0-6):**
0 (Absent) = No disorganised communication.
1 (Questionable) = Mild awkward/hesitant phrasing, overuse of jargon; typically self-reported only; self-corrects
readily; minimal distress/interference.
2 (Mild) = Slightly vague/over-elaborate speech or idiosyncratic word use; may be self-report only; usually aware
and self-corrects; mild impact.
3 (Moderate) = Observable incorrect word use, brief circumstantiality, or occasional irrelevant topics (off-track
but returns); self-correction variable; some impact on clarity.
4 (Marked) = Prolonged circumstantial speech, difficulty goal-directing sentences, sudden pauses; requires some
structuring/redirection; distress/interference present.
5 (Severe but not psychotic) = Tangentiality (never gets to point), loosening of associations, or blocking; needs
frequent prompts/structuring; clear interference with functioning/communication.
6 (Psychotic and very severe) = Derailment/completely loose associations, unintelligible or internally inconsistent
speech, severe blocking/echolalia; not responsive to redirection; marked interference.

**Frequency (0-6):**
0 = Absent
1 = Less than one day a month
2 = One day a month to two days a week - < 1 hour/day
3 = One day a month to two days a week - > 1 hour/day OR 3--6 days/week - < 1 hour/day
4 = 3--6 days/week - > 1 hour/day OR daily - < 1 hour/day
5 = Daily - > 1 hour/day OR several times/day
6 = Continuous

---

### Output format
Return only JSON:

{
  "severity": <integer 0-6>,
  "frequency": <integer 0-6>,
  "summary": "<1 paragraph (3-5 sentences) summarizing symptoms
  alongside the severity and frequency for a psychiatrist's initial assessment>"
}
```
